# Phylogeny and evolution of Müllerian mimicry in aposematic *Dilophotes*: evidence for advergence and size-constraints in evolution of mimetic sexual dimorphism

**DOI:** 10.1038/s41598-018-22155-6

**Published:** 2018-02-27

**Authors:** Michal Motyka, Lucie Kampova, Ladislav Bocak

**Affiliations:** Laboratory of Molecular Systematics, Faculty of Science, Palacky University, 17. listopadu 50, 771 46 Olomouc, Czech Republic

## Abstract

Multiple patterns and intraspecific polymorphism should not persist in mutualistic Müllerian systems due to purifying and frequency-dependent selection, but they are commonly identified in nature. We analysed molecular phylogeny and reconstructed dispersal history of 58 species of *Dilophotes* (Coleoptera: Lycidae) in Asia. *Dilophotes* colonized the Great Sundas and Malay Peninsula where they joined extensive mimetic communities of net-winged beetles. We identified the brightly bi-coloured males and females which adverged on five occasions to different autochthonous models. This is the first described case of Müllerian sexual dimorphism based on sex-specific body size. We propose that the constraint, i.e. the conservative sexual size dimorphism, forced the unprofitable prey to such complex adaptation in a multi-pattern environment. Although mimetic sexual dimorphism has frequently evolved in *Dilophotes*, a single pattern has been maintained by both sexes in multiple closely related, sympatrically occurring species. Some patterns may be suboptimal because they are rare, crudely resemble co-mimics, or are newly evolved, but they persist in Müllerian communities for a long time. We assume that failure to closely resemble the most common model can increase the diversity of large Müllerian communities and produce mimetic dimorphism.

## Introduction

The Müllerian mimicry model describes an anti-predatory strategy of unprofitable prey, which expresses a warning signal to potential predators^1^. Numerous studies predicted the mutualistic evolution of a single warning pattern and strong selection against rare forms in Müllerian systems^1–5^. In contrast with the simplicity of Müller’s original model, common deviations can be identified in nature. Müllerian systems contain multiple aposematic patterns^2^, imperfect mimics^6–8^, and intraspecific polymorphism^9–13^. Many adaptive processes have been proposed to explain the unpredicted phenomena, among them the selection for a general pattern^7,14^, relaxed selection due to the cognitive capacity of predators^8,14,15^, and the high costs of error preventing attacks on all crudely matched individuals^8,14^. Further, the existence of Müllerian multi-pattern complexes can be explained by locally relaxed predation due to the absence of predators^16^, adaptive neophobia^17^, and micro-habitat partition of both predator and prey populations^18–20^. These adaptive factors can explain survival of imperfect mimics as well as presence of multiple patterns in a single locality due to relaxed negative selection. The non-adaptive hypotheses include drift and constraints including an evolutionary time-lag^8,21,22^, but without any further evidence. These non-adaptive factors are responsible for the continuous origins of new, divergent and imperfect mimics and they define time needed for the exclusion of some aposematic patterns. Even under strong purifying selection, some co-mimics remain only crudely similar and imperfect mimicry is often observed^6–8^, possibly due to the interplay between all above described factors.

As an additional factor in the evolution of mimicry, we need to consider if all unprofitable prey develop the common aposematic pattern through convergence as originally hypothesized^1^ or advergence^23^. Advergence assumes that a new, usually rare aposematically coloured prey unilaterally adopts a previously established Müllerian aposematic signal. Advergence can start with the dispersal of the originally allopatric unprofitable prey, represented by a low number of individuals, to an area with an extensive autochthonous Müllerian system. The additional member joins the ring with a time delay which depends on selection against rare forms^24–26^ and the capability to adopt locally dominant aposematic pattern^24–26^. We may suppose that dynamic changes of distribution can lead to the contact between two unpalatable preys with different aposematic signal. The mathematical models and laboratory experiments^1,27^ have not considered the constraints in evolution of multi-pattern Müllerian mimetic communities. Dated phylogenies can potentially identify the delayed evolution of pigments and mimetic patterns and identify the gradual increase in numbers of species sharing individual mimetic patterns. Such phylogenies have been constructed for some clades of Müllerian mimics^28,29^, but never for net-winged beetles (Coleoptera: Lycidae).

Here, we study the phylogeny of *Dilophotes*, one of several dozens of aposematically coloured net-winged beetle genera occurring in eastern and southeastern Asia^30,31^. All net-winged beetles are unprofitable^32^ and their protection can be easily identified in the field as they pungently smell and spontaneously bleed when disturbed (Fig. S1E). These unprofitable beetles form complex multi-pattern Müllerian systems, especially in the tropical rainforest of South East Asia, where hundreds of aposematically coloured net-winged beetles occur. *Dilophotes* represents a promising model group for the study of mimicry. Its origin was dated to the Upper Cretaceous^30^, the genus is widespread, and currently there are 61 formally described species (Fig. S2). Due to limited sclerotization of their body, all net-winged beetles are poor dispersers and they only slowly expand their ranges^33^. The net-winged beetle fauna shows high turnover in individual mountain ranges and volcanoes and most species are known only from a single mountain range^34^. Additionally, net-winged beetles are common only in humid mountain habitats. As a consequence, highly diverse communities typically occur within a very small altitudinal span, usually from 1000–1800 m above sea level, and the turnover among individual mountain ranges and volcanoes is almost complete^11,35^. The communities of net-winged beetles are composed of a relatively high number of species, which supposedly share a long common evolutionary history^30^. The co-mimics of *Dilophotes* include *Libnetis* (Libnetini; ~300 species in the Great Sundas and the Malay peninsula), *Plateros* (Platerodini; ~500 spp.), *Cautires*, *Xylobanus* (Metriorrhynchini; ~600 spp.), and several smaller genera with ~300 species in total. Most of them are more common than *Dilophotes* and occur in high numbers over the entire region.

We set the origins of mimetic patterns of *Dilophotes* in a phylogenetic context and investigated the adoption process of various patterns and the variability of evolutionary strategies. Specifically, we focused on the absence of some colour patterns in subclades of *Dilophotes* and dated the origins of distinct colour patterns. Our study identifies the multiple origins of unique sexual colour dimorphism and sexual size dimorphism as a factor potentially responsible for the evolution of such a complex mimetic system.

## Results and Discussion

**Figure 1:**
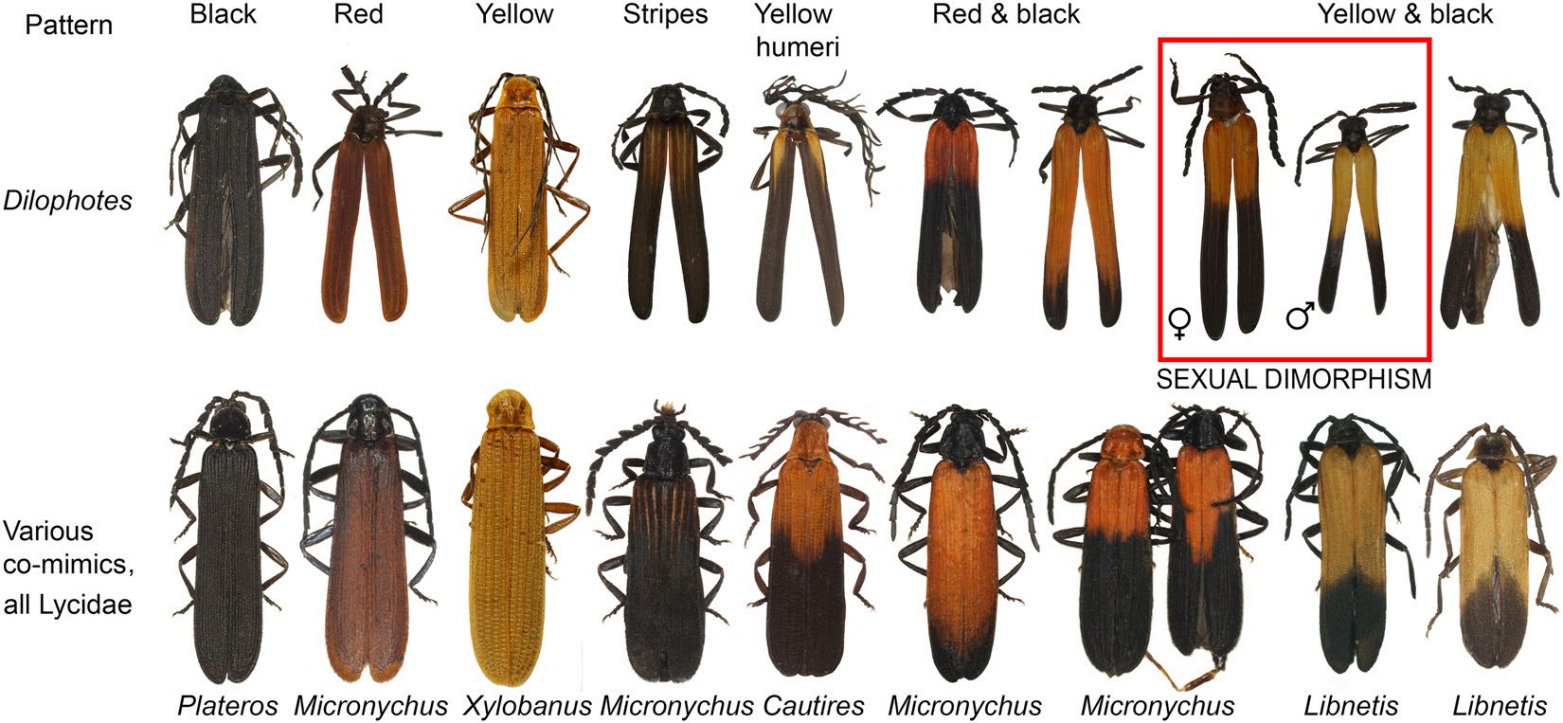
Aposematically coloured net-winged beetles. Photographs © Authors.

**Figure 2:**
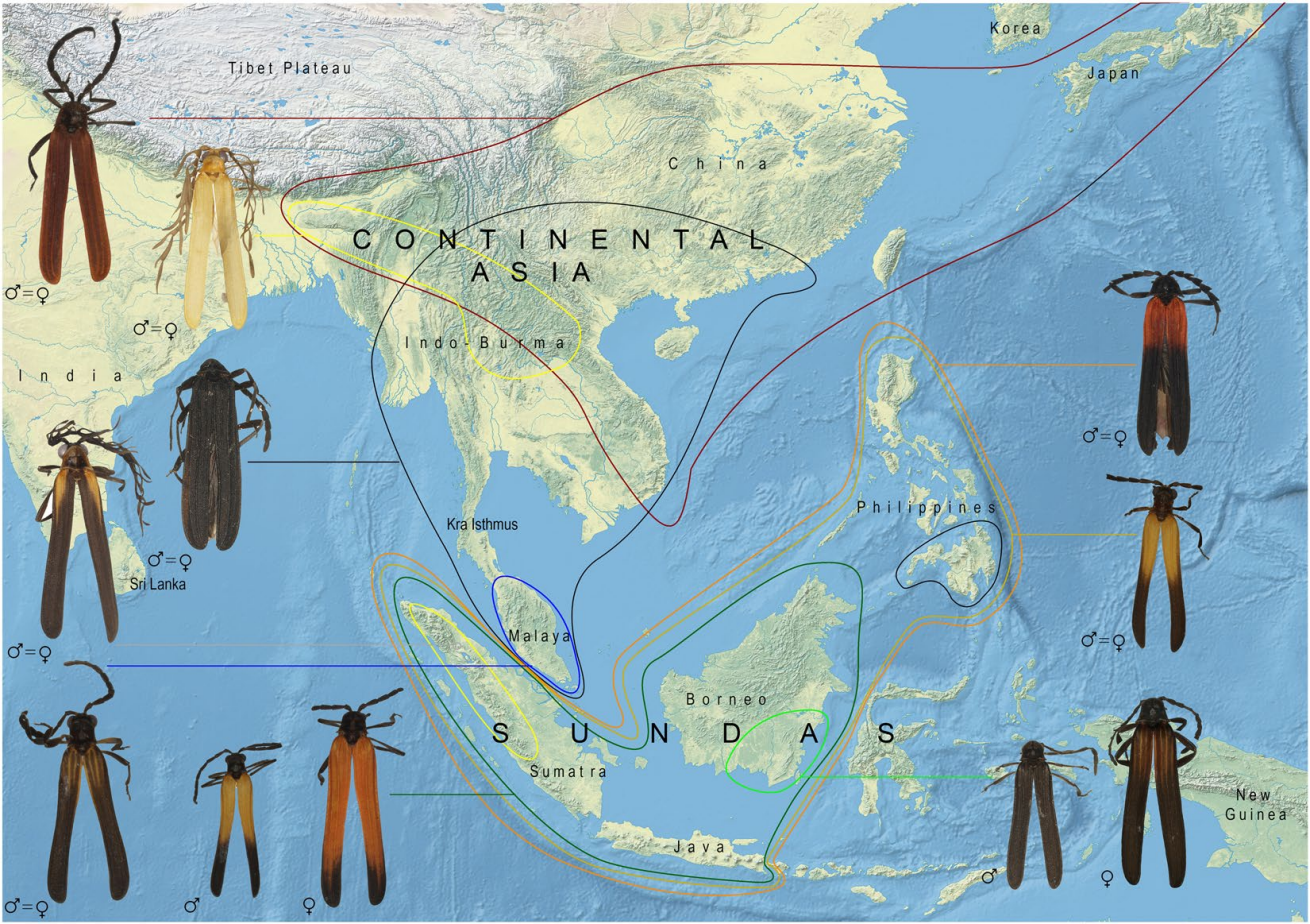
Distribution of mimetic patterns of *Dilophotes*. The map was downloaded from Natural Earth server (http://www.naturalearthdata.com) and edited using Adobe Photoshop CS6 (http://www.adobe.com/products/photoshop.html).

**Figure 3:**
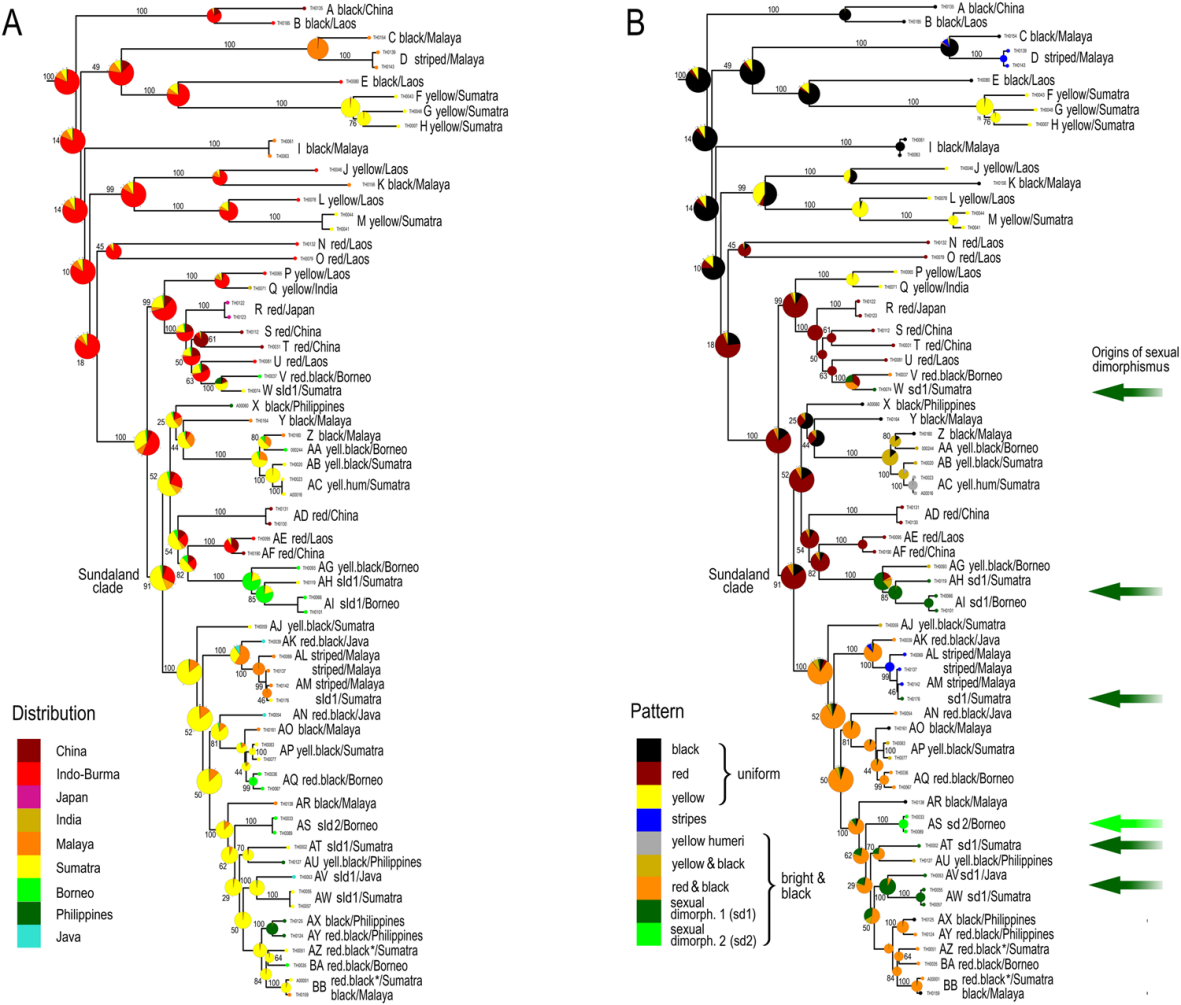
Phylogenetic reconstruction of ancestral states: (**A**) geographic origin; (**B**) colour patterns.

**Figure 4:**
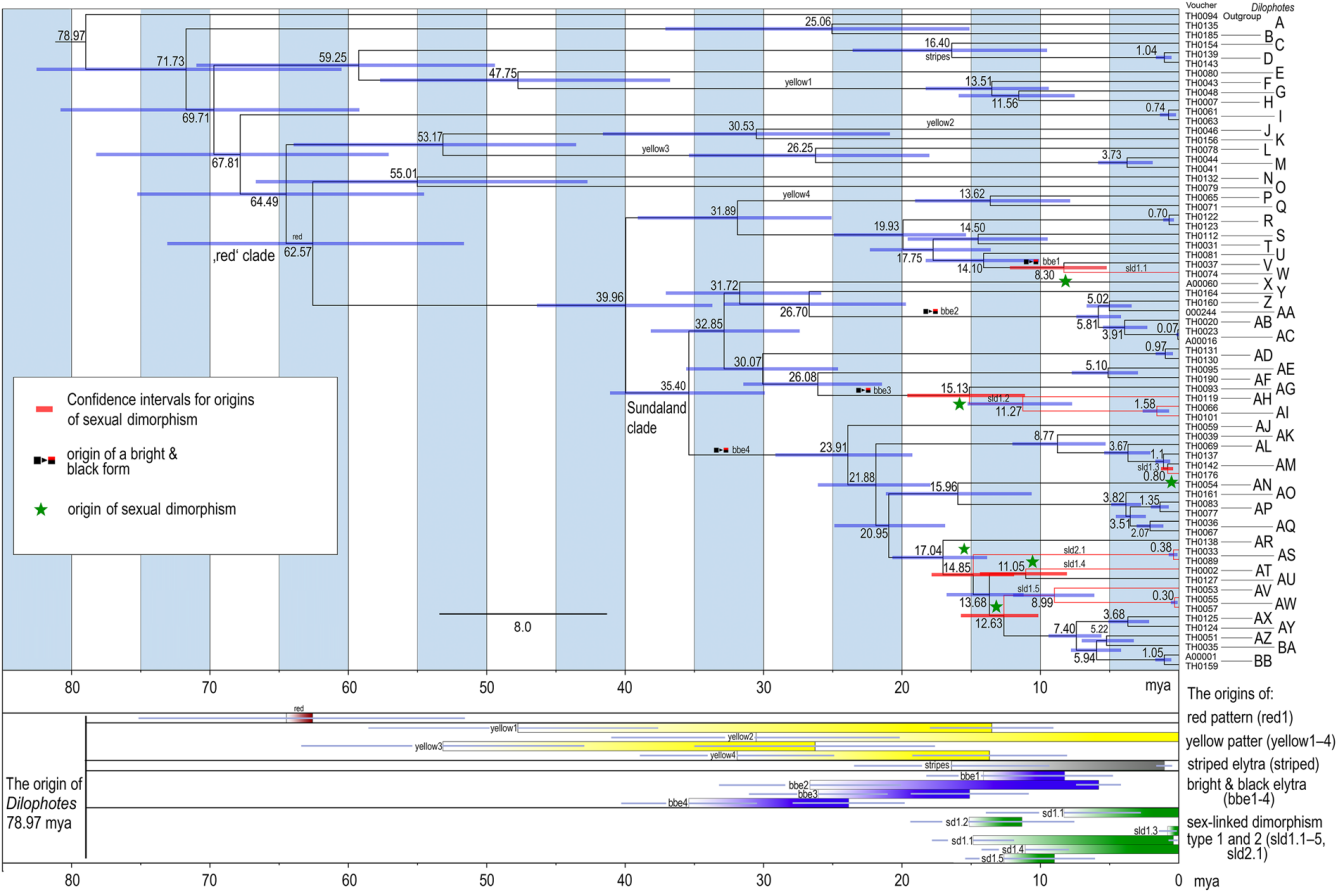
Dated tree with mean times of origin and 95% confidence intervals.

**Figure 5:**
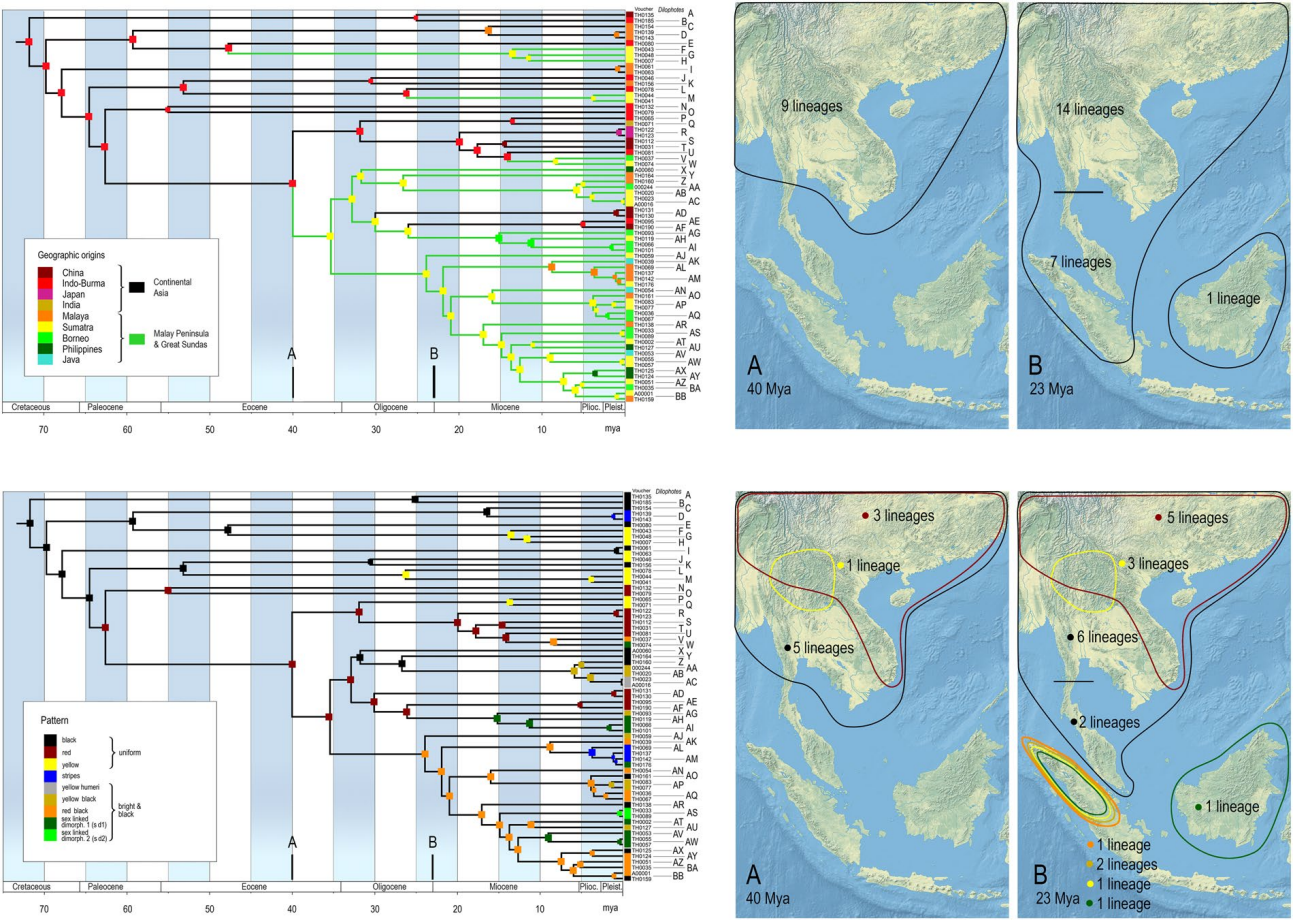
(**A**) Distribution of *Dilophotes* in the Upper Eocene and at the Oligocene/Miocene boundary; (**B**) Presence and distribution of aposematic patterns in the Upper Eocene and at the Oligocene/Miocene boundary. The map was downloaded from Natural Earth server (http://www.naturalearthdata.com) and edited using Adobe Photoshop CS6 (http://www.adobe.com/products/photoshop.html).

Our dataset of unprofitable aposematic *Dilophotes* (Coleoptera: Lycidae; Fig. 1) contains 58 species from continental Asia, the Sunda Islands, and the Philippines (Table S1, Figs 2, S2). The origin of the *Dilophotes* clade is estimated to have been in Indo-Burma ~79 million years ago (mya) with dispersals to the south several times in the last 35 my (Figs 3A–5A). All species have geographically restricted ranges, typically a single island or a mountain system on the continent with several endemic species syntopically occurring in each locality (Fig. S1B). Dispersal events are rare and species coexist in the same area for long periods.

### Diversity and origins of patterns in Müllerian rings

The observed colour patterns were classified into seven discrete types shared by both sexes: the patterns ‘black’, ‘red’, and ‘yellow’ have uniformly coloured upper sides of the body, i.e. pronotum and elytra. Further, we identified ‘striped’, ‘yellow humeri’, ‘yellow & black’, and ‘red & black’ patterns, which have bi-coloured elytra (Figs 1 and 2). Surprisingly, we identified two combinations of different patterns in conspecific males and females. These sexually dimorphic patterns are represented by ‘yellow & black’ males and ‘red & black’ females (sexual dimorphism type 1, ‘sd1’) or ‘black’ males and ‘striped’ females (‘sd2’). There are multiple syntopically occurring aposematically coloured species of other net-winged beetles. They belong to several different subfamilies and tribes and are distantly related^30^ (Fig. 1).

The colouration of *Dilophotes* is diverse. Up to five pattern shifts were inferred in an ancestor-descendent lineage, i.e., the sequence of splits from the deepest diversification event to the splits between extant species (Fig. 3). The number of patterns increased steadily over time; almost half of the species pairs contains terminals belonging to different mimetic patterns (as species pair we consider two extant species which share a single most common recent ancestor which is not shared by any other extant species in the analysis; most species pairs represent very closely related species, see Figs 3, S4; it is possible that some additional species will be discovered which will be more related to one of considered species than to the other, see Fig. S1A for comparison of formally described and analysed diversity).

**Figure 6:**
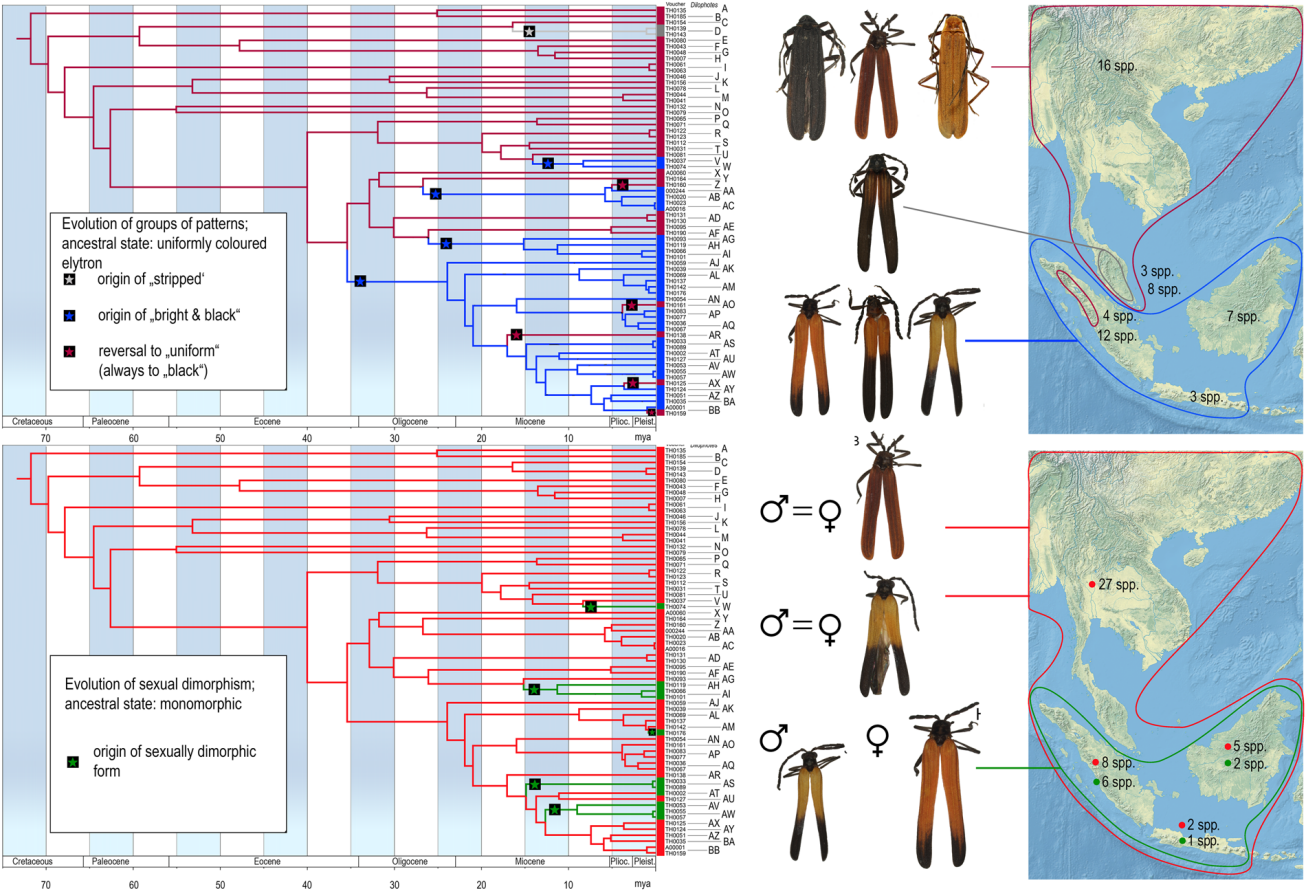
(**A**) Evolution of ‘uniform’ and ‘bright & black’ patterns; (**B**) Evolution of sexually dimorphic patterns. The map was downloaded from Natural Earth server (http://www.naturalearthdata.com) and edited using Adobe Photoshop CS6 (http://www.adobe.com/products/photoshop.html).

Mimetic patterns evolved gradually. The reconstruction of colour patterns shows that a black colouration was already present in Indo-Burma ~79 mya (Figs 3–6). The ‘black’ type does not represent a typical aposematic pattern as many palatable insects are black-coloured and most unprofitable insects are brightly coloured. We suppose that the body shape and size presumably are the salient optical warning signals^27^ (compare the body shape of the Zygaenidae moth and *Lyropaeus* net-winged beetle in Fig. S1A,B). We assume that distinctiveness has an aposematic function when prey is observed against a clear sky because the high contrast makes it easily recognisable^36,37^ (Fig. S3E). The ancestrally ‘black’ clades gave rise to the ‘yellow’ pattern in Indo-Burma and Sumatra. The distinctiveness of the ‘yellow’ pattern is conditional^38^: these beetles are very conspicuous if sitting on the upper side of a leaf due to the high contrast^37^ with dark green leaves under the tropical forest canopy. Conversely, they are much less conspicuous, at least to a human observer, if an individual sits on the bottom side and is observed against a clear sky (unpublished field observation). This pattern is displayed by all brightly uniformly coloured *Dilophotes* south of the Kra Isthmus. The single origin of a red pigment occurred ~63 mya in Indo-Burma, 16 mya after the origin of *Dilophotes* (Fig. 3). Red-coloured Dictyopterinae, an old cosmopolitan lineage^31^ with the stem age of the clade of red-coloured taxa inferred at 90.1 ± 2.2 mya^30^, could be considered a potential model for uniformly ‘red’ *Dilophotes*. The ‘red’ pattern is currently limited to its ancestral area and was lost by all lineages dispersing to the south, i.e. to the Malay Peninsula and the Great Sundas. The first origin of the ‘bright & black’ forms was dated to 35.4 mya, long after the origin of the ‘red’ pattern and three other origins are substantially younger (8.3–14.1 mya, Figs 4–6). The ‘bright & black’ pattern is dominant in the Sundaland net-winged beetles (Fig. 1, the dominance is based on the proportion of ‘bright & black’ individuals in the studied sample and on the observation in the field, see Fig. S1B and Table S4). The bi-coloured pattern never evolved in the ancestral area of the “red” pattern and we suppose that its evolution was forced by the presence of many species of bi-coloured net-winged beetles in the Greater Sunda Islands where *Dilophotes* dispersed (Fig. 2A).

Information about the pigments of elateroid beetles is scarce^39^, but we may hypothesize, based on information from other beetles, that melanins are responsible for black and yellow and pterins for red colouration. The ‘yellow’ pattern can be easily produced when the concentration of melanins is decreased and four origins of the ‘yellow’ pattern were identified (Fig. 3). Conversely, the acquisition of a different pigment is necessary for red colouration and we identified a single origin of the ‘red’ pattern (Fig. 3).

The ‘bright & black’ pattern evolved four times, always within the ancestrally ‘red’ clade, and never in two other deeply rooted ancestrally ‘black’ lineages also occurring in Sumatra (Fig. 2). The number of analysed species is low, therefore the scenario of gradual acquisition of aposematic patterns should be demonstrated in further lineages. The evolution of a bright and contrasting patterns, generally considered as more effective^40–44^ and easily memorable^45,46^, seems to be a hurdle on the trajectory to an effective signal. Although field experiments testing the effectiveness of individual pattern signalling unpalatability are not available for *Dilophotes*, or any other net-winged beetle, we can compare the observed evolution of aposematic patterns with suggestions from experiments with artificial prey^47^. If similar processes are hypothesised, then the ‘uniform’ patterns are weaker, i.e. less conspicuous or less memorable, than ‘bright & black’ patterns^36,37,44,47–49^.

### Origin of sexual dimorphism

**Figure 7:**
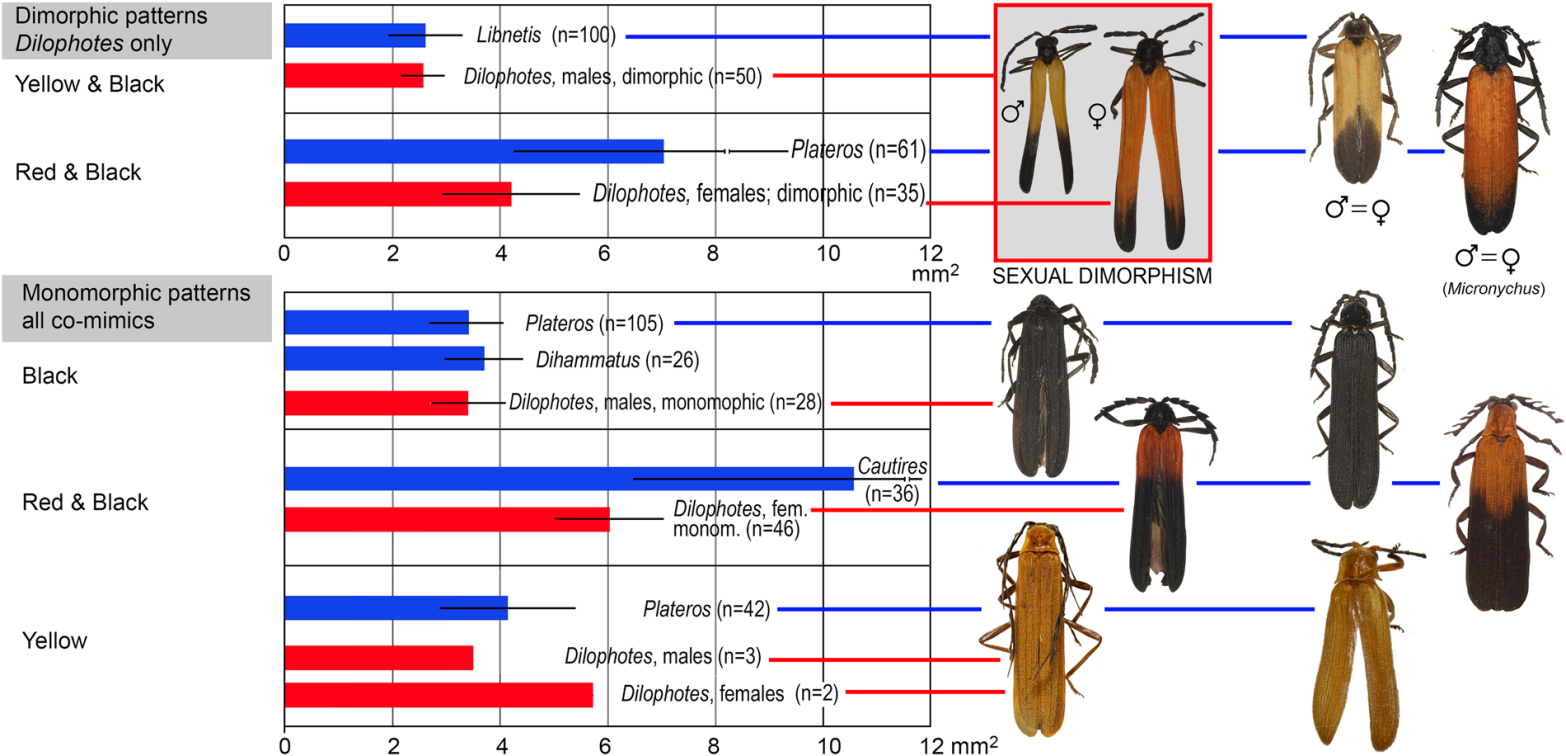
Average body size of *Dilophotes* and their co-mimics (n = number of measured individuals).

The theory predicts monomorphism for unprofitable mimetic prey, and the known cases of intraspecific polymorphism have involved geographical and micro-habitat forms^9^ as in the cases of polymorphic syntopically occurring Müllerian mimics in butterflies^10^ and also in recently reported net-winged beetles^11–13^. In *Dilophotes*, we report intraspecific sexual dimorphism with males and females resembling different syntopically occurring models (Fig. 1). The sex-dimorphic form ‘sd1’ evolved five times (15.0–0.8 mya) within the ancestrally ‘bright & black’ clades in the Sundas (Figs 2, 3). The complexity of copying different models by each sex is indicated by the close similarity of males, but only the crude similarity of females to all putative models (Fig. 1). The small-bodied males (elytron, 5.02 ± 0.80 mm^2^) closely resemble the body size of *Libnetis* and *Plateros*, most of which are yellow and black if brightly coloured (Fig. 1). The females of *Dilophotes* are larger (6.31 ± 1.02 mm^2^, Table S2) and resemble ‘red & black’ *Micronychus*, *Cautires*, *Plateros* etc., but their similarity is imperfect, as they are regularly smaller than their models (Fig. 7). This system shows that *Dilophotes* has constrained body size and both sexes cannot closely copy the large body size of the dominant ‘bright & black’ syntopically occurring net-winged beetles. Nevertheless, *Dilophotes* never adopted a general pattern^14,15^, i.e. an intermediate imperfect pattern which can be assigned to several distinct sympatric patterns and we observe the incentive of males to be closely similar to a local model^1,5^ (Figs 1, 7). Additionally, similar intraspecific sexual dimorphism ‘sd2’ was identified in a single species from southern Borneo. Only a few individuals are available from a single area, therefore, these divergent male and female phenotypes cannot be studied in detail (Figs 2, 3B).

We inferred advergence as the principal mode of building Müllerian rings with the roles of a ‘mimic’ and a ‘model’, despite both being equally protected^23^. Such a process is robustly supported in sexually dimorphic species. The alternative explanation is simultaneous convergence of three species which are not brightly coloured and as a result the first species adopts ‘red & black’ pattern, the second species adopts ‘yellow & black’ pattern and the third species becomes dimorphic with ‘red & black’ females and ‘yellow & black’ males. We consider such process improbable. Comparing a low proportion of *Dilophotes* in mimetic communities and their age with the diversity, age, and distribution of putative models^31^, we assume that the first dispersing *Dilophotes* already met aposematically coloured autochthonous models and were selected for similarity with them^50–52^.

### Diversity of aposematic patterns in Müllerian rings of *Dilophotes*

We noted that closely related lineages of aposematically coloured *Dilophotes* (Fig. 2) often belong to different mimetic rings even when they are the members of a single community. We assumed that all these species were in close interaction with other unprofitable net-winged beetles in their communities. As stated above, the net-winged beetles are common in Asia only in lower montane forests; they have small ranges limited to mountain ranges and individual volcanoes, and the turnover among localities is almost complete (Fig. S2). Additionally, the different mimicry patterns were commonly recorded together in net-winged beetle aggregations in the field (e.g. the aggregation described in the Supplementary Text). Although we cannot exclude some microhabitat fragmentation as in butterflies^18–20^, all individuals included in this study were collected together with other syntopically occurring net-winged beetles displaying various aposematic patterns.

As an example we can describe origins and distribution of monomorphic and dimorphic species. We identified seven sex-dimorphic species (Fig. 2) and thirteen monomorphic species ‘bright & black’ species in the Greater Sundas. All these species are closely related (Fig. 3) and belong to the Sundaland clade of morphologically uniform species. The recorded aposematic patterns include: (A) Both sexes adverged to the small-bodied ‘yellow & black’ pattern. The model, i.e. *Libnetis* spp., is small-bodied (Fig. 7) and we assumed that only males and small-bodied females of *Dilophotes* are perfect mimics. As net-winged beetles do not feed in the adult stage^31^, small-bodied females may produce a lower mass of eggs^53^ and advergence to small-bodied co-mimics may incur costs^14^. (B) Both sexes adopted the ‘red & black’ pattern. All males and even the largest females are much smaller than their ‘red & black’ co-mimics and they are imperfect mimics (Fig. 7). Therefore, both sexes will potentially suffer a higher predation rate^5^. (C) The males adverged to the ‘yellow & black’ pattern and the females to the ‘red & black’ pattern (body-size defined sexual dimorphism). The females can increase their body size, i.e. avoid lower fecundity predicted under scenario A; the male is a perfect mimic and does not experience a negative effect on fitness. Such a strategy, although not perfect due to the imperfect similarity of females, evolved on five occasions (Fig. 7).

The delayed evolution or inaccessibility of a local pattern in Müllerian systems has not been considered in theoretical models nor has it been shown by experiments, although such factors can substantially affect the structure of Müllerian communities. The adoption of the ‘red’ pattern took millions of years; most ancestrally black clades never evolved the contrasting ‘bright & black’ pattern which evolved only in ancestrally ‘red’ subclades (bce1–4, Fig. 3). Similarly, sexual dimorphism evolved only in the ancestrally ‘bright & black’ clade with a 10–20 my delay after the colonization of the Sundas. Five origins of ‘sd1’ in the last 15 mya, including one very recent, support the continual adoption of sexual dimorphism (Figs 3–6). Conversely, many ancestrally ‘bright & black’ *Dilophotes* have remained monomorphic, despite their presence in an area with ‘bright & black’ co-mimics for about 13 my and undoubtedly being at least periodically exposed to a similar selective pressure. We suppose that they were following a suboptimal strategy being unable to closely resemble the most common syntopic mimicry model or having lower fecundity due to small female body size. Further, one species evolved a new, unique colour pattern, ‘yellow humeri’, which does not match any co-mimic (Fig. 1), probably profiting only from some level of protection in a multi-pattern community^43,54^ or due to some level of neophobia among predators^55^. One species evolved the sex-dimorphic pattern ‘sd2’ (Fig. 3B). Hence, a large part of *Dilophotes* missed the opportunity to join the dominant ‘red & black’ Müllerian ring containing hundreds of species, which should provide the strongest protection^56^. The described distribution of aposematic pattern opens the question why closely related species living within a single community follow different strategies. We might consider the mosaic structure of the environment and dynamic changes of the environment and niche structure (climate, altitudinal changes, stochastic fluctuations in abundance of co-mimetics, etc.). These factors are adaptive. Similarly, non-adaptive constraints may affect the structure of Müllerian communities: e.g., the delayed origin of colour pigments or different body size resulting in imperfect resemblance. We assume that these processes might be responsible for the high number of aposematic patterns in a single community. Our study is limited by *Dilophotes* as a model, a single geographical region and rareness of these Müllerian mimics. Our hypotheses are based on empirical observation and should be tested by theoretical models which should include a high number of interacting species forming extensive networks^57,58^ affected by dynamically changing selective processes due to fluctuating abundance of individual members. The models should also consider different rates of evolution of at least several traits important in signalling unpalatability, e.g., the body size, shape, and colouration.

## Conclusion

Sexual dimorphism has never been predicted by theoretical models of Müllerian mimicry, which do not consider constraints or the long-term character of adaptive processes. In some *Dilophotes*, both sexes are aposematically coloured, but they follow different models. The described system supports the adaptive character of Müllerian mimicry^2^, but instead of coevolution suggests dominant advergence in the gradually expanding multi-pattern communities. Under such conditions, the described evolution of the unique sexual dimorphism is strong evidence for the role of different male and female body sizes during the adoption of syntopically occurring mimicry patterns. The inferred delayed evolution of individual mimetic patterns (Figs 4, 5B, 6A,B), adoptions of uncommon and weak mimetic signals^37,47,48,58,59^, the evolution of a new pattern^1^, and their persistence (Figs 2, 4) suggest the possibility that multiple factors affect the coexistence of aposematic patterns in Müllerian communities.

## Methods

### Sampling and morphology

Over 700 adult *Dilophotes* were collected from 28 localities across continental Asia, the Great Sundas, and the Philippines from 1991–2012 (Fig. S2). The species were identified algorithmically using DNA data and tested using morphological divergence (putative species are marked with uppercase codes and merged putative species, i.e. which did not differ morphologically, with lowercase codes added). Altogether 196 individuals representing all colour patterns and 58 species (Table S3) from the sampled localities were analysed using previously reported protocols^30^ (Table S4); 29 additional samples represented outgroups (Table S1). The length of the elytron (EL) and width at humeri (WH) were measured and the surface of the elytron (EL*WH) was counted for all analysed specimens and sympatrically occurring lycid co-mimics to estimate sexual size dimorphism. The surface was used as a proxy for the size-dependent strength of an aposematic signal (Table S2, Fig. 3B). Colouration of the pronotum and elytra was recorded and colour patterns were grouped into nine arbitrary categories further used in the reconstruction of ancestral states (Table S2, Figs 1, 2 and S3).

### Phylogenetic analyses

The sequences of three mtDNA fragments were produced: 780 bp of the large subunit ribosomal mtDNA (*rrnL*) with tRNA-Leu and a fragment of NADH dehydrogenase 1 (*nad1*); 1100 bp fragment of cytochrome oxidase 1 mtDNA (*cox1*), tRNA-Leu gene, and cytochrome oxidase 2 (*cox2*); and 1180 bp of NADH dehydrogenase 5 mtDNA (*nad5*) and adjacent tRNAs (fragments are referred as *rrnL*, *cox1*, and *nad5* further, Table S5).

The fragments were aligned using MAFFT 7.2 (Q-INS-I algorithm)^60^ under default parameters and the protein coding fragments were checked for reading frames. The concatenated matrices were analysed under the likelihood criterion using RAXML 7.2.3^61,62^ with the model identified by jModelTest 2^63^ using the AIC criterion. The dataset was partitioned by genes and codon positions (Table S5) and analysed with 1,000 bootstrap replicates under the GTRCAT substitution model^61^. The dataset was also analysed using MrBayes 3.1.2^64^. The MCMC was set with independent parameters for the same partitions as above, under the general time reversible model with a category of invariant sites and gamma distributed rates (GTR + I + G). Four chains were run simultaneously for 6 × 10^7^ generations, with trees being sampled every 10,000 generations; all fragments were partitioned and unlinked. The pre-stationary phase was identified using Tracer 1.6^65^.

Due to the poor taxonomic state, species were identified algorithmically using the general mixed Yule-coalescent (GMYC) model^66^ and the results were evaluated using the morphology of the male genitalia. The GMYC approach applies the threshold time to separate species determined from an ultrametric tree. We used the algorithm implemented in the SPLITS package for R (http://r-forge.r-project.org/projects/splits/; 2009)^66^. The ultrametric tree was produced using the all-data matrix (Table S1) and the same settings as in the BEAST analysis of the pruned dataset (below).

### Estimation of divergence times

The pruned and complete trees were dated using a Bayesian approach implemented in BEAST 1.8.2^67^. The first analysis was performed with one representative per species and population (Table S1) and set to HKY + I + G proposed as the second-best model (the analyses using the GTR + I + G model did not converge)^63^, Relaxed Clock: Uncorrelated Lognormal, the Tree Model to a Speciation: Birth-Death Process and were set to 50 million generations with sampling every 2,500 generations with the first 25% of trees discarded as burn-in. The genes and codon positions were partitioned (Table S5) and each partition was provided with its own parameters. Because the fossil record is poor for net-winged beetles, we used information on the mtDNA rate in Coleoptera to calibrate topology. We used a mean rate of 0.0115 substitutions per site per million years per lineage, subs/s/my/l) for *cox1*^68^, 0.0167 subs/s/my/l for *nadh5*^69^, and 0.0054 subs/s/my/l for *rrnL*^70^. The conclusions depend more on the inference of the gradual evolution of the individual mimicry patterns than on detailed timing, therefore we considered the calibration sufficient for the purposes of this study. We set the ucld.mean with a normal distribution. The best topology recovered from the ML analysis (Fig. S1) was fixed by the guiding tree and switching off trees operators during analysis^67^. Convergence was assessed in Tracer 1.6^65^ and the first 1.25 × 10^7^ generations were set as burn-in.

### Ancestral area and colour pattern reconstructions

Discrete phylogeographic reconstructions were performed using the ‘Discrete traits’ function in BEAST 1.8.2^68^ on the pruned dataset. To reconstruct the ‘Ancestral area’ character, each species was assigned to one of the following states: China, Laos/Thailand, Japan, Northern India, Malay Peninsula, Sumatra, Borneo, the Philippines, or Java. Three reconstructions of colour pattern evolution were performed in the same way with (A) ‘elytral colouration’ states: ‘uniform’, ‘striped’, and ‘bright & black’, (B) ‘mimetic pattern’: monomorphic and dimorphic; and (C) ‘colouration pattern’: ‘black’, ‘red’, ‘yellow’, ‘stripes’, ‘yellow humeri’, ‘yellow & black’, ‘red & black’, ‘sexual dimorphic type 1’ (‘sd1’), and ‘sexual dimorphic type 2’ (‘sd2’) (Fig. 1A–J). All analyses were run under settings described at https://beast-classic.googlecode.com/files/ARv2.0.1.pdf for 50 million generations with sampling every 2,500 generations and the first 25% of trees discarded as burn-in. The ESS values were checked in Tracer 1.6^65^ and the maximum credibility tree was generated in TREEANNOTATOR 1.8.1 when a minimum 500 ESS value was obtained^68^.

## Electronic supplementary material

Supplements
