# Supplementary material for: Phylogeny and evolution of Müllerian mimicry in aposematic Dilophotes: evidence for advergence and size-constraints in evolution of mimetic sexual dimorphism
Source: Sci Rep. 2018 Feb 27;8:3744. doi: 10.1038/s41598-018-22155-6 (PMC5829258; doi:10.1038/s41598-018-22155-6)
Supplement: Supplementary file 1 — Supplements [file 41598_2018_22155_MOESM1_ESM.pdf]

Phylogeny and evolution of Müllerian mimicry in aposematic *Dilophotes*: evidence for advergence and size-constraints in evolution of mimetic sexual dimorphism

Michal Motyka, Lucie Kampova & Ladislav Bocak

The list of Supplementary materials

Supplementary Texts. Biology of net-winged beetles (Coleoptera: Lycidae).
Field observations, mimicry pattern classification, and distribution.

Supplementary Table S1. The list of sequenced specimens with GenBank Accession Numbers and geographic origins.
Supplementary Table S2. Overview of color patterns and body measurements for all analysed specimens.
Supplementary Table S3. The delineation of biological species based on morphology and GMYC analysis.
Supplementary Table S4. Primers used for PCR amplifications and sequencing.
Supplementary Table S5. The length of DNA fragments, the numbers of informative characters in datasets, and partitions.
Supplementary Table S6. The node probability values for the reconstruction of the evolution of the monomorphic and dimorphic aposematic patterns. Tree nodes as defined in the Figure S6.
Supplementary Table S7. The node probability values for the reconstruction of uniform and bicolored elytra.
Supplementary Table S8. The node probability values for the reconstruction of individual aposematic patterns.
Supplementary Table S9. The node probability values for the reconstruction of the ancestral areas.
Supplementary Figure S1. Color patterns of net-winged beetles in the Malay Peninsula and Papua.
Supplementary Figure S2. A – Sampled localities in Malaya, the Sundaland, and Philippines with the numbers of formally described and recorded species; B – Distribution of color patterns and species in Malaya, the Sundaland, and the Philippines.
Supplementary Figure S3. Color patterns of net-winged beetles in the Sundaland.
Supplementary Figure S4. The maximum likelihood phylogenetic hypothesis of *Dilophotes* inferred from the MAFFT aligned dataset.
Supplementary Figure S5. The Bayesian phylogenetic hypothesis of *Dilophotes* inferred from the MAFFT aligned dataset.
Supplementary Figure S6. The numbers designating splits in the pruned tree.
Supplementary Figure S7. The Bayesian phylogenetic reconstruction of ancestral states. A – uniform and bright & black elytral patterns; B – sexually monomorphic and dimorphic aposematic patterns.

Supplementary Text.

Biology of net-winged beetles (Coleoptera: Lycidae).

Lycidae is an elateroid beetle family with 4300 species distributed worldwide, but most groups are known from wet humid tropics[1]. The tribe Dilophotini contains 61 validly described species occurring in the eastern part of the Palearctic region and throughout the Oriental region[2,3]. The classification of the tribe is in complete chaos and identification is almost impossible when many types are females, which do not have usable diagnostic characters. Therefore, the species are delimited in the current study using mtDNA sequences and individual species are designated by letters (Tab. S3)

Whole net-winged beetle family is characterized by very uniform biology. The larvae have mandibles split in two blades and they are able to take only liquids from decaying organic material. Larvae live cryptically in upper layers of soil rich in organic material, in decaying roots in soil or in rotten wood[4] (Fig. 3F). The adults of most species avoid sunny places and remain under canopy, where they sit on leaves or sometimes decaying wood. The slow and cumbersome flight is characteristic for all lycids and instead escape reaction all lycids are unpalatable and aposematically coloured. Few genera, especially in arid regions, visit flowers to take nectar, but most net-winged beetles, including *Dilophotes*, remain under canopy sitting on leaves and do not take any food in adult stage[5].

Most net-winged beetles are brightly colored (Fig. S2A) and their colour patterns are shared by various species of beetles (Pyrochoridae, Oedemetidae, Chrysomelidae, *etc.*) and by some moths (e.g. Zygaenidae, Lepidoptera; Fig. S1B). The bright coloration is very common in Metriorrhynchini (*Metanoeus*, *Cautires* and *Xylobanus* in the Oriental region, Figs S1C, S3A–B), Calochromini (Figs 1L, P–Q), some Platerodini (red & black *Plateros* in the Oriental region, brightly yellow & black *Plateros* in most of its range) and *Libnetis* (yellow or yellow and black patterns in the Oriental region). Uniform dark coloration is common in some groups, typically Lycinae: Platerodini and Libnetinae (Fig. S3C–E). Some net-winged beetles are monochromatically but brightly coloured: brightly red, yellow or alternatively, some net-winged beetles from Indo-Burma and China have dark-red uniform colour of elytra. Even species which are not brightly colored, closely resemble each other or unrelated insects in the similar body-size and shape (Fig. 3A–B). We suppose that the shape and size represent a signal of their unpalatability, especially when an individual is sitting on the bottom side of the leaf and is observed against light coming from above (Fig. S3E).

Dilophotini are similarly uniformly black, dark-red or yellow colored in the northern part of their range and Malay mountains. A high number of species from the Great Sundas and Philippines have bright & black coloured elytra with brightly coloured humeri and the black apical part.

Information on unpalatability of Lycidae is limited[6,7]. We identified typical smell across all major beetles lineages of net-winged beetles during our field research. The disturbed individuals are bleeding from various body parts including elytra and the membranes between segments in legs, antennae and thorax (Fig. S1F).

Field observations, mimicry pattern classification, and distribution.

The material was collected in 28 localities across Eastern and South East Asia (Fig. S2A). A large part of material for this study was taken from an aggregated group of net-winged beetles at the margin of the mountain forest in the Gunung Sibayak area (Northern Sumatra, Fig. S1, Tab. S1, coordinates: 3°13'13"N 98°29'58"E). The aggregation occupied about 600 sq. m and some 900 net-winged beetle specimens were collected on this limited place. Additionally, several dozens of net-winged beetles were collected in a larger area during a several day stay. The observed aggregation is exceptionally large, but aggregations are typical for aposematically coloured net-winged beetles. Multiple taxa were represented in this community: *Dilophotes* (~400 males, ~50 females, representing 7 species, tribe Dilophotini), *Libnetis* (~300 spec., 4 species, Libnetinae: Libnetini), *Plateros* (~120 spec., 5 species, Lycinae: Platerodini), *Cautires* (56 spec., 6 spec., Lycinae: Metriorrhynchini), *Micronychus* (8 spec., 2 species, Lycinae: Calochromini), and *Xylobanus* (8 spec., 2 species, Lycinae: Metriorrhynchini). Altogether four aposematic patterns were found in this single aggregation (Fig. 1, Tab. S3). Further similarly coloured individuals were collected in various localities in Sumatra and additional few dozens of net-winged beetle species belonged to various tribes and genera, some of them are illustrated in Fig. 1. Further material was collected throughout the Oriental and Eastern Palearctic regions, always syntopically with numerous species of similarly colored net-winged beetles. *Dilophotes* was among the less common lycids in all localities, always representing <10% of collected net-winged beetle specimens except the aggregation described above.

Altogether, nine color patterns changing across the studied areas were identified in *Dilophotes* (Fig. 1). The 'yellow/black' and 'red/black' aposematic patterns form widespread mimicry rings in the Great Sundas. Additional patterns have restricted occurrence: 'striped' occur in lowlands of Sumatra, Borneo and the Malay Peninsula, 'black' pattern in the high elevations of the Malay Peninsula, e.g. Cameron Highlands in Pahang, and in some places in Southern Borneo. The pattern 'yellow' is less common and is represented by a limited number of species from the genera *Dilophotes*, *Plateros*, *Xylobanus*, and *Libnetis* in lower elevations of Oriental region and in Indo-Burma. The pattern 'yellow humeri' is known only in a single species from a single locality in Northern Sumatra. Figure 2A shows the distribution of colour patterns on the phylogenetic tree. The pattern 'yellow/black', i.e., uniform dark body coloration and yellow to brown humeri and dark colored rest of elytra with the body size 3–7 mm, is common in *Dilophotes*, *Scarelus*, *Plateros*, and *Libnetis*. The pattern 'red/black' occur seldom in *Scarelus* and *Plateros*. Regularly, the species having medium-sized to large body (5–17 mm) have brightly orange or red humeri and black elytral apexes. This colour pattern is common in *Cautires*, *Xylobanus*, *Metanoeus*, *Micronychus*, and some Platerodini.

References

1. Bocak, L. & Bocakova, M. Phylogeny and classification of the family Lycidae (Insecta: Coleoptera). *Annales Zoologici* **58**, 695–720 (2008)
2. Kleine R. *Lycidae. Pars 128*. In W. Junk *Coleopterorum Catalogus auspiciis et auxilio,* S. Schenkling. Berlin (1933)
3. Bic, V. Review of the genus *Dilophotes* Waterhouse (Coleoptera: Lycidae) of the Palaearctic Region and Indochina. *Zootaxa* **59**, 1–26 (2002)
4. Bocak, L. & Matsuda, K. Review of the immature stages of the family Lycidae (Insecta: Coleoptera). *Journal of Natural History* **37**, 1463–1507 (2003)
5. Bocak, L. & Bocakova, M. *Family Lycidae*. In: Leschen R. A. B. & Beutel R. G. (Eds) Handbook of Zoology. Part 38, Vol. 2. Morphology and Systematics (Elateroidea, Bostrichiformia, Cucujoidea). Walter de Gruyter, Berlin, New York (2010)
6. Moore, B. P. & Brown W. V. Identification of warning odour components, bitter principles and antifeedants in an aposematic beetle - *Metriorrhynchus rhipidium* (Coleoptera: Lycidae). *Ins. Biochem.* **15**, 493–499 (1981)
7. Eisner, T., Schroeder, F. C., Snyder, N., Grant, J. B., Aneshansley, D. J., Utterback, D., Meinwald, J., & Eisner, M. Defensive chemistry of lycid beetles and of mimetic cerambycid beetles that feed on them. *Chemoecology* **18**, 109–119 (2008)

Supplementary Table S1. The list of sequenced specimens with GenBank Accession Numbers and geographic origins.

Voucher Number	Genus	Subfamily: tribe	Geographic origin	GenBank Access. Numb./Fragment*** *rrnL+ tRNA-Leu+ nad1*	*cox1+ tRNA-Leu+ cox2*	*nad5+ tRNA-Phe+ tRNA-Glu+ tRNA-Ser*
**Outgroups***						
UPOLA00583	*Libnetis* sp.	Libnetinae	China	KT752081	KT751758	KT751915
UPOLA00587	*Libnetis* sp.	Libnetinae	Malaysia	KT752085	KT751762	KT751919
UPOLA00589	*Libnetis* sp.	Libnetinae	Sumatra	KT752087	KT751764	KT751921
UPOLA00591	*Libnetis* sp.	Libnetinae	Laos	KT752089	KT751766	KT751923
UPOLL01008	*Libnetis* sp.	Libnetinae	Malaysia	DQ181031	DQ181253	DQ181407
UPOLTH0082	*Libnetis* sp.	Libnetinae	Sumatra, S. Barat, Gn. Merapi	KJ404971	KJ405170	KJ405320
UPOLTH0084	*Libnetis* sp.	Libnetinae	Sumatra, S. Barat, Gn. Merapi	KJ404973	-	KJ405322
UPOLTH0085	*Libnetis* sp.	Libnetinae	Sumatra, S. Barat, Gn. Merapi	KJ404974	-	KJ405323
UPOLTH0086	*Libnetis* sp.	Libnetinae	Sumatra, S. Barat, Gn. Merapi	KJ404975	-	KJ405324
UPOLTH0087	*Libnetis* sp.	Libnetinae	Sumatra, S. Barat, Gn. Merapi	KJ404976	-	KJ405325
UPOLTH0149	*Libnetis* sp.	Libnetinae	Malay Pen., Kelantan, L. Pandrak	KJ405037	-	-
UPOLTH0150	*Libnetis* sp.	Libnetinae	Malay Pen., Kelantan, L. Pandrak	KJ405038	KJ405232	-
UPOLTH0045	*Plateros* sp.	Lycinae: Platerodini	Indonesia, Sumatra	KJ404936	KJ405137	-
UPOLTH0058	*Plateros* sp.	Lycinae: Platerodini	Indonesia, Sumatra	KJ404949	KJ405150	-
UPOLTH0094	*Macrolycus* sp.	Lycinae: Macrolycini	Laos, Hua Phan, Saleui	KJ404983	KJ405178	KJ405332
UPOLVK0733	*Atelius* sp.	Lycinae: Ateliini	China	KT752146	KT751822	KT751975
UPOLVM0004	*Scarelus* sp.	Lycinae: Ateliini	Malaysia	HM451000	HM451040	HM451209
UPOLVM0012	*Scarelus* sp.	Lycinae: Ateliini	Sumatra	HM451006	HM451047	HM451217
UPOLVM0019	*Scarelus* sp.	Lycinae: Ateliini	Borneo	HM451012	HM451051	-
UPOLVM0021	*Scarelus* sp.	Lycinae: Ateliini	Borneo	HM451014	HM451053	HM451222
UPOLVM0022	*Scarelus* sp.	Lycinae: Ateliini	Borneo	HM451015	HM451054	HM45122
UPOLVM0049	*Scarelus* sp.	Lycinae: Ateliini	Philippines	HM451035	HM451076	HM451248
UPOLYL0217	*Ponyalis* sp.	Lycinae: Lyponiini	China	-	KJ650408	-
UPOLYL0259	*Ponyalis* sp.	Lycinae: Lyponiini	Japan	-	KJ650438	-
UPOLYL0314	*Lyponia* sp.	Lycinae: Lyponiini	China	-	KJ650493	-
UPOLYL0409	*Ponyalis* sp.	Lycinae: Lyponiini	China	-	KJ650437	-

Ingroup

Voucher Number	Genus	Species Code	Geographic origin	GenBank Accession Numbers, mtDNA *rrnL*	*cox1*	*nad5*
UPOLTH0135	*Dilophotes*	A	China, Hainan, Jianfengling	KJ405024	KJ405218	-
UPOLTH0185	*Dilophotes*	B	Laos, Hua Phan, Phu Phan	KJ405073	KJ405266	KJ405393
UPOLTH0186	*Dilophotes*	B	Laos, Hua Phan, Phu Phan	KJ405074	KJ405267	KJ405394
UPOLTH0187	*Dilophotes*	B	Laos, Hua Phan, Phu Phan	KJ405075	-	KJ405395
UPOLTH0188	*Dilophotes*	B	Laos, Hua Phan, Phu Phan	KJ405076	KJ405268	KJ405396
UPOLTH0189	*Dilophotes*	B	Laos, Hua Phan, Phu Phan	KJ405077	KJ405269	KJ405397
UPOLTH0154	*Dilophotes*	C	Malay Peninsula, Johor, Kotatinggi	KJ405042	KJ405236	-
UPOLTH0139	*Dilophotes*	D	Malay Peninsula, Johor, Kotatinggi	KJ405028	KJ405222	KJ405362
UPOLTH0141	*Dilophotes*	D	Malay Peninsula, Kelantan, Gua Musang	KJ405030	KJ405224	-
UPOLTH0143	*Dilophotes*	D	Malay Peninsula, Kelantan, Gua Musang	KJ405032	KJ405226	KJ405363
UPOLTH0144	*Dilophotes*	D	Malay Peninsula, Kelantan, Gua Musang	KJ405033	KJ405227	KJ405364
UPOLTH0080	*Dilophotes*	E	Laos, Hua Phan, Ban Saluei	KJ404969	KJ405168	KJ405318
UPOLTH0042	*Dilophotes*	F	Sumatra, S. Barat, Pasaman, Gn. Talamau	-	KJ405134	-
UPOLTH0043	*Dilophotes*	F	Sumatra, S. Barat, Pasaman, Gn. Talamau	KJ404934	KJ405135	-
UPOLTH0048	*Dilophotes*	G	Sumatra, S. Utara, Brastagi, Gn. Sinnabung	KJ404939	KJ405140	KJ405301
UPOLA00008	*Dilophotes*	H	Sumatra, S. Utara, Brastagi, Gn. Sibayak	KJ404893	KJ405086	KJ405275
UPOLTH0007	*Dilophotes*	H	Sumatra, S. Utara, Brastagi, Gn. Sibayak	KJ404908	KJ405101	KJ405284
UPOLTH0008	*Dilophotes*	H	Sumatra, S. Utara, Brastagi, Gn. Sibayak	KJ404909	KJ405102	KJ405285
UPOLTH0009	*Dilophotes*	H	Sumatra, S. Utara, Brastagi, Gn. Sibayak	KJ404910	KJ405103	KJ405286
UPOLTH0015	*Dilophotes*	H	Sumatra, S. Utara, Brastagi, Gn. Sibayak	KJ404915	KJ405107	-
UPOLTH0061	*Dilophotes*	I	Malay Pen., Pahang, Rd Ipoh-Kg. Raja	KJ404951	-	KJ405302
UPOLTH0063	*Dilophotes*	I	Malay Peninsula, Pahang, Tanah Rata	KJ404952	-	KJ405303
UPOLTH0046	*Dilophotes*	J	Laos, Bolikhamsai, Ban Nape	KJ404937	KJ405138	-
UPOLTH0038	*Dilophotes*	K	Malay Peninsula, Pahang, Gn. Jasar	KJ404930	KJ405130	-
UPOLTH0068	*Dilophotes*	K	Malay Peninsula, Pahang, Tanah Rata	KJ404957	KJ405156	KJ405307
UPOLTH0136	*Dilophotes*	K	Malay Peninsula, Pahang, Gn. Jasar	KJ405025	KJ405219	-
UPOLTH0147	*Dilophotes*	K	Malay Pen., Kelantan, Ladang Pandrak	-	KJ405230	-
UPOLTH0151	*Dilophotes*	K	Malay Pen., Kelantan, Ladang Pandrak	KJ405039	KJ405233	-
UPOLTH0153	*Dilophotes*	K	Malay Peninsula, Johor, Kotatinggi	KJ405041	KJ405235	-
UPOLTH0156	*Dilophotes*	K	Malay Peninsula, Johor, Kotatinggi	KJ405044	KJ405238	KJ405366

UPOLTH0078	*Dilophotes*	L	Laos, Hua Phan, Ban Saluei	KJ40467	KJ405166	KJ405316
UPOLTH0041	*Dilophotes*	M	Sumatra, S. Barat, Pasaman, Gn. Talamau	KJ404933	KJ405133	-
UPOLTH0044	*Dilophotes*	M	Sumatra, S. Barat, Lake Maninjau	KJ404935	KJ405136	-
UPOLTH0132	*Dilophotes*	N	Laos, Hua Phan, Phu Phan	KJ405021	KJ405215	KJ405360
UPOLTH0079	*Dilophotes*	O	Laos, Hua Phan prov., Ban Saluei	KJ404968	KJ405167	KJ405317
UPOLTH0133	*Dilophotes*	O	Laos, Hua Phan prov., Phu Phan	KJ405022	KJ405216	-
UPOLTH0134	*Dilophotes*	O	Laos, Hua Phan prov., Phu Phan	KJ405023	KJ405217	-
UPOLTH0064	*Dilophotes*	P	Thailand, Chiangmai, Doi Pui	KJ404953	-	KJ405304
UPOLTH0065	*Dilophotes*	P	Thailand, Chiangmai, Doi Pui	KJ404954	KJ405153	KJ405305
UPOLTH0070	*Dilophotes*	Q	India, Arunachal, Dirang	KJ404959	KJ405158	KJ405308
UPOLTH0071	*Dilophotes*	Q	India, Arunachal, Dirang	KJ404960	KJ405159	KJ405309
UPOLTH0072	*Dilophotes*	Q	India, Arunachal, Dirang	KJ404961	KJ405160	KJ405310
UPOLTH0073	*Dilophotes*	Q	India, Arunachal, Dirang	KJ404962	KJ405161	KJ405311
UPOLTH0102	*Dilophotes*	Q	India, Arunachal, Dirang	KJ404991	KJ405186	KJ405340
UPOLTH0103	*Dilophotes*	Q	India, Arunachal, Dirang	KJ404992	KJ405187	KJ405341
UPOLTH0104	*Dilophotes*	Q	India, Arunachal, Dirang	KJ404993	KJ405188	KJ405342
UPOLTH0105	*Dilophotes*	Q	India, Arunachal, Dirang	KJ404994	KJ405189	KJ405343
UPOLTH0106	*Dilophotes*	Q	India, Arunachal, Dirang	KJ404995	KJ405190	KJ405344
UPOLTH0107	*Dilophotes*	Q	India, Arunachal, Dirang	KJ404996	KJ405191	KJ405345
UPOLTH0108	*Dilophotes*	Q	India, Arunachal, Dirang	KJ404997	KJ405192	KJ405346
UPOLTH0109	*Dilophotes*	Q	India, Arunachal, Dirang	KJ404998	KJ405193	KJ405347
UPOLTH0110	*Dilophotes*	Q	India, Arunachal, Dirang	KJ404999	KJ405194	KJ405348
UPOLTH0111	*Dilophotes*	Q	India, Arunachal, Dirang	KJ405000	KJ405195	KJ405349
UPOLTH0122	*Dilophotes*	R	Japan, Tokushima, Tsurugisan	KJ405011	KJ405205	KJ405353
UPOLTH0123	*Dilophotes*	R	Japan, Tokushima, Akaboshiyama	KJ405012	KJ405206	KJ405354
UPOLTH0112	*Dilophotes*	S	China, Taiwan, Nantou county	KJ405001	KJ405196	KJ405350
UPOLTH0031	*Dilophotes*	T	China, Shaanxi, Qin Mts.	KJ404925	KJ405123	-
UPOLTH0081	*Dilophotes*	U	Laos, Hua Phan prov., Ban Saleui	KJ404970	KJ405169	KJ405319
UPOLTH0037	*Dilophotes*	V	Borneo, Sabah, Gn. Emas	KJ404929	KJ405129	-
UPOLTH0049	*Dilophotes*	W	Sumatra, Jambi, Kersik Tua, Gn. Tujuh	KJ404940	KJ405141	-
UPOLTH0050	*Dilophotes*	W	Sumatra, Jambi, Kersik Tua, Gn. Tujuh	KJ404941	KJ405142	-
UPOLTH0074	*Dilophotes*	W	Sumatra, Jambi, Kersik Tua, Gn. Kerinci	KJ404963	KJ405162	KJ405312
UPOLTH0075	*Dilophotes*	W	Sumatra, Jambi, Kersik Tua, Gn. Kerinci	KJ404964	KJ405163	KJ405313
UPOLA00060	*Dilophotes*	X	Philippines, Negros, Mt. Canlaon	KC538740	KC538359	KC538552

UPOLTH0148	*Dilophotes*	Y	Malay Peninsula, Kelantan, Ladang Pandrak	KJ405036	KJ405231	-
UPOLTH0152	*Dilophotes*	Y	Malay Peninsula, Pahang, Gn. Beremban	KJ405040	KJ405234	KJ405365
UPOLTH0158	*Dilophotes*	Y	Malay Peninsula, Johor, Kotatinggi	KJ405046	KJ405240	-
UPOLTH0164	*Dilophotes*	Y	Malay Peninsula, Kelantan, Ladang Pandrak	KJ405052	KJ405246	KJ405372
UPOLTH0160	*Dilophotes*	Z	Malay Peninsula, Kelantan, Ladang Pandrak	KJ405048	KJ405242	KJ405368
UPOL000244	*Dilophotes*	AA	Borneo, Sabah, Gn. Emas	DQ181214	DQ180992	DQ181368
UPOLA00007	*Dilophotes*	AB	Sumatra, S. Utara, Brastagi, Gn. Sibayak	KJ404892	KJ405085	-
UPOLTH0014	*Dilophotes*	AB	Sumatra, S. Utara, Brastagi, Gn. Sibayak	KJ404914	KJ405106	-
UPOLTH0020	*Dilophotes*	AB	Sumatra, S. Utara, Brastagi, Gn. Sibayak	KJ404920	KJ405112	KJ405292
UPOLTH0021	*Dilophotes*	AB	Sumatra, S. Utara, Brastagi, Gn. Sibayak	KJ404921	KJ405113	KJ405293
UPOLTH0022	*Dilophotes*	AB	Sumatra, S. Utara, Brastagi, Gn. Sibayak	KJ404922	KJ405114	-
UPOLTH0114	*Dilophotes*	AB	Sumatra, S. Utara, Brastagi, Gn. Sibayak	KJ405003	KJ405198	KJ405351
UPOLTH0118	*Dilophotes*	AB	Sumatra, S. Utara, Brastagi, Gn. Sibayak	KJ405007	KJ405201	-
UPOLTH0120	*Dilophotes*	AB	Sumatra, S. Utara, Brastagi, Gn. Sibayak	KJ405009	KJ405203	-
UPOLTH0121	*Dilophotes*	AB	Sumatra, S. Utara, Brastagi, Gn. Sibayak	KJ405010	KJ405204	-
UPOLA00016	*Dilophotes*	AC	Sumatra, S. Utara, Brastagi, Gn. Sibayak	KJ404901	KJ405257	-
UPOLTH0023	*Dilophotes*	AC	Sumatra, S. Utara, Brastagi env.	KJ404923	KJ405115	KJ405294
UPOLTH0129	*Dilophotes*	AD	China, Hainan, Limushan, summit	KJ405018	KJ405212	-
UPOLTH0130	*Dilophotes*	AD	China, Hainan, Limushan, summit	KJ405019	KJ405213	KJ405359
UPOLTH0131	*Dilophotes*	AD	China, Hainan, Limushan env.	KJ405020	KJ405214	-
UPOLTH0095	*Dilophotes*	AE	Laos, Hua Phan, Saluei	KJ404984	KJ405179	KJ405333
UPOLTH0190	*Dilophotes*	AF	China, Guangdong, Dadong Shan	KJ405078	KJ405270	KJ405398
UPOLTH0093	*Dilophotes*	AG	Borneo, Kalimantan Selatan, Loksado	KJ404982	KJ405177	KJ405331
UPOLA00011	*Dilophotes*	AH	Sumatra, S. Utara, Brastagi, Gn. Sibayak	KJ404896	KJ405089	-
UPOLA00012	*Dilophotes*	AH	Sumatra, S. Utara, Brastagi, Gn. Sibayak	KJ404897	KJ405090	-
UPOLTH0006	*Dilophotes*	AH	Sumatra, S. Utara, Brastagi, Gn. Sibayak	KJ404907	KJ405100	KJ405283
UPOLTH0119	*Dilophotes*	AH	Sumatra, S. Utara, Brastagi, Gn. Sibayak	KJ405008	KJ405202	KJ405352
UPOLTH0175	*Dilophotes*	AH	Sumatra, S. Utara, Brastagi, Gn. Sibayak	KJ405063	KJ405257	KJ405383
UPOLTH0088	*Dilophotes*	AI	Borneo, Kalimantan Selatan, Loksado	KJ404977	KJ405172	KJ405326
UPOLTH0091	*Dilophotes*	AI	Borneo, Kalimantan Selatan, Loksado	KJ404980	KJ405175	KJ405329
UPOLTH0096	*Dilophotes*	AI	Borneo, Kalimantan Selatan, Loksado	KJ404985	KJ405180	KJ405334
UPOLTH0097	*Dilophotes*	AI	Borneo, Kalimantan Selatan, Loksado	KJ404986	KJ405181	KJ405335
UPOLTH0099	*Dilophotes*	AI	Borneo, Kalimantan Selatan, Loksado	KJ404988	KJ405183	KJ405337
UPOLTH0100	*Dilophotes*	AI	Borneo, Kalimantan Selatan, Loksado	KJ404989	KJ405184	KJ405338

UPOLTH0101	*Dilophotes*	AI	Borneo, Kalimantan Selatan, Loksado	KJ404990	KJ405185	KJ405339
UPOLTH0066	*Dilophotes*	AI	Borneo, Sabah, Gn. Emas	KJ404955	KJ405154	-
UPOLTH0059	*Dilophotes*	AJ	Sumatra, S. Barat, Muara Sako	KJ404950	KJ405151	-
UPOLTH0060	*Dilophotes*	AJ	Sumatra, S. Barat, Muara Sako	-	KJ405152	-
UPOLTH0039	*Dilophotes*	AK	Java, J. Tenggah, Gn. Lawu, Sarangan	KJ404931	KJ405131	-
UPOLTH0040	*Dilophotes*	AK	Java, J. Tenggah, Gn. Lawu, Sarangan	KJ404932	KJ405132	-
UPOLTH0069	*Dilophotes*	AL	Malay Peninsula, Pahang, Tanah Rata	KJ404958	KJ405157	-
UPOLA00003	*Dilophotes*	AM	Sumatra, S. Utara, Brastagi, Gn. Sibayak	KJ404888	KJ405081	KJ405272
UPOLA00004	*Dilophotes*	AM	Sumatra, S. Utara, Brastagi, Gn. Sibayak	KJ404889	KJ405082	KJ405273
UPOLA00006	*Dilophotes*	AM	Sumatra, S. Utara, Brastagi, Gn. Sibayak	KJ404891	KJ405084	KJ405274
UPOLA00009	*Dilophotes*	AM	Sumatra, S. Utara, Brastagi, Gn. Sibayak	KJ404894	KJ405087	KJ405276
UPOLA00014	*Dilophotes*	AM	Sumatra, S. Utara, Brastagi, Gn. Sibayak	KJ404899	KJ405092	KJ405277
UPOLTH0003	*Dilophotes*	AM	Sumatra, S. Utara, Brastagi, Gn. Sibayak	KJ404904	KJ405097	KJ405280
UPOLTH0010	*Dilophotes*	AM	Sumatra, S. Utara, Brastagi, Gn. Sibayak	KJ404911	KJ405104	KJ405287
UPOLTH0011	*Dilophotes*	AM	Sumatra, S. Utara, Brastagi, Gn. Sibayak	KJ404912	-	-
UPOLTH0017	*Dilophotes*	AM	Sumatra, S. Utara, Brastagi, Gn. Sibayak	KJ404917	KJ405109	-
UPOLTH0019	*Dilophotes*	AM	Sumatra, S. Utara, Brastagi, Gn. Sibayak	KJ404919	KJ405111	KJ405291
UPOLTH0027	*Dilophotes*	AM	Sumatra, S. Utara, Brastagi, Gn. Sibayak	-	KJ405119	-
UPOLTH0028	*Dilophotes*	AM	Sumatra, S. Utara, Brastagi, Gn. Sibayak	-	KJ405120	-
UPOLTH0047	*Dilophotes*	AM	Sumatra, S. Utara, Brastagi, Gn. Sinnabung	KJ404938	KJ405139	KJ405300
UPOLTH0113	*Dilophotes*	AM	Sumatra, S. Utara, Brastagi, Gn. Sibayak	KJ405002	KJ405197	-
UPOLTH0167	*Dilophotes*	AM	Sumatra, S. Utara, Brastagi, Gn. Sibayak	KJ405055	KJ405249	KJ405375
UPOLTH0168	*Dilophotes*	AM	Sumatra, S. Utara, Brastagi, Gn. Sibayak	KJ405056	KJ405250	KJ405376
UPOLTH0171	*Dilophotes*	AM	Sumatra, S. Utara, Brastagi, Gn. Sibayak	KJ405059	KJ405253	KJ405379
UPOLTH0172	*Dilophotes*	AM	Sumatra, S. Utara, Brastagi, Gn. Sibayak	KJ405060	KJ405254	KJ405380
UPOLTH0173	*Dilophotes*	AM	Sumatra, S. Utara, Brastagi, Gn. Sibayak	KJ405061	KJ405255	KJ405381
UPOLTH0176	*Dilophotes*	AM	Sumatra, S. Utara, Brastagi, Gn. Sibayak	KJ405064	KJ405258	KJ405384
UPOLTH0179	*Dilophotes*	AM	Sumatra, S. Utara, Brastagi, Gn. Sibayak	KJ405067	KJ405261	KJ405387
UPOLTH0181	*Dilophotes*	AM	Sumatra, S. Utara, Brastagi, Gn. Sibayak	KJ405069	KJ405263	KJ405389
UPOLTH0182	*Dilophotes*	AM	Sumatra, S. Utara, Brastagi, Gn. Sibayak	KJ405070	KJ405264	KJ405390
UPOLTH0184	*Dilophotes*	AM	Sumatra, S. Utara, Brastagi, Gn. Sibayak	KJ405072	KJ405265	KJ405392
UPOLTH0140	*Dilophotes*	AM	Malay Peninsula, Kelantan, Gua Musang	KJ405029	KJ405223	-
UPOLTH0142	*Dilophotes*	AM	Malay Peninsula, Kelantan, Gua Musang	KJ405031	KJ405225	-
UPOLTH0145	*Dilophotes*	AM	Malay Peninsula, Kelantan, Gua Musang	KJ405034	KJ405228	-

UPOLTH0137	*Dilophotes*	AM	Malay Peninsula, Johor, Kotatinggi	KJ405026	KJ405220	-
UPOLTH0054	*Dilophotes*	AN	Java, S. Barat, Puncak Pass, Cipanas	KJ404945	KJ405146	-
UPOLTH0146	*Dilophotes*	AO	Malay Peninsula, Kelantan, Ladang Pandrak	KJ405035	KJ405229	-
UPOLTH0161	*Dilophotes*	AO	Malay Peninsula, Kelantan, Ladang Pandrak	KJ405049	KJ405243	KJ405369
UPOLTH0162	*Dilophotes*	AO	Malay Peninsula, Kelantan, Ladang Pandrak	KJ405050	KJ405244	KJ405370
UPOLTH0163	*Dilophotes*	AO	Malay Peninsula, Kelantan, Ladang Pandrak	KJ405051	KJ405245	KJ405371
UPOLTH0077	*Dilophotes*	AP	Sumatra, Jambi, Kersik Tua, Gn. Kerinci	KJ404966	KJ405165	KJ405315
UPOLTH0083	*Dilophotes*	AP	Sumatra, S. Barat, Gn. Merapi	KJ404972	KJ405171	KJ405321
UPOLTH0032	*Dilophotes*	AQ	Borneo, Sabah, Gn. Emas	-	KJ405124	-
UPOLTH0034	*Dilophotes*	AQ	Borneo, Sabah, Gn. Emas	-	KJ405126	-
UPOLTH0036	*Dilophotes*	AQ	Borneo, Sabah, Gn. Emas	KJ404928	KJ405128	-
UPOLTH0067	*Dilophotes*	AQ	Borneo, Sabah, Gn. Emas	KJ404956	KJ405155	KJ405306
UPOLTH0138	*Dilophotes*	AR	Malay Peninsula, Johor, Kotatinggi	KJ405027	KJ405221	KJ405361
UPOLTH0033	*Dilophotes*	AS	Borneo, Kalimantan Tenggah, Muara Teweh	KJ404926	KJ405125	-
UPOLTH0089	*Dilophotes*	AS	Borneo, Kalimantan Selatan, Loksado	KJ404978	KJ405173	KJ405327
UPOLTH0090	*Dilophotes*	AS	Borneo, Kalimantan Selatan, Loksado	KJ404979	KJ405174	KJ405328
UPOLTH0092	*Dilophotes*	AS	Borneo, Kalimantan Selatan, Loksado	KJ404981	KJ405176	KJ405330
UPOLTH0098	*Dilophotes*	AS	Borneo, Kalimantan Selatan, Loksado	KJ404987	KJ405182	KJ405336
UPOLA00002	*Dilophotes*	AT	Sumatra, S. Utara, Brastagi, Gn. Sibayak	KJ404887	KJ405080	-
UPOLA00005	*Dilophotes*	AT	Sumatra, S. Utara, Brastagi, Gn. Sibayak	KJ404890	KJ405083	-
UPOLA00010	*Dilophotes*	AT	Sumatra, S. Utara, Brastagi, Gn. Sibayak	KJ404895	KJ405088	-
UPOLA00013	*Dilophotes*	AT	Sumatra, S. Utara, Brastagi, Gn. Sibayak	KJ404898	KJ405091	-
UPOLA00015	*Dilophotes*	AT	Sumatra, S. Utara, Brastagi, Gn. Sibayak	KJ404900	KJ405093	-
UPOLTH0001	*Dilophotes*	AT	Sumatra, S. Utara, Brastagi, Gn. Sibayak	KJ404902	KJ405095	KJ405278
UPOLTH0002	*Dilophotes*	AT	Sumatra, S. Utara, Brastagi, Gn. Sibayak	KJ404903	KJ405096	KJ405279
UPOLTH0004	*Dilophotes*	AT	Sumatra, S. Utara, Brastagi, Gn. Sibayak	KJ404905	KJ405098	KJ405281
UPOLTH0005	*Dilophotes*	AT	Sumatra, S. Utara, Brastagi, Gn. Sibayak	KJ404906	KJ405099	KJ405282
UPOLTH0012	*Dilophotes*	AT	Sumatra, S. Utara, Brastagi, Gn. Sibayak	KJ404913	KJ405105	KJ405288
UPOLTH0016	*Dilophotes*	AT	Sumatra, S. Utara, Brastagi, Gn. Sibayak	KJ404916	KJ405108	KJ405289
UPOLTH0018	*Dilophotes*	AT	Sumatra, S. Utara, Brastagi, Gn. Sibayak	KJ404918	KJ405110	KJ405290
UPOLTH0024	*Dilophotes*	AT	Sumatra, S. Utara, Brastagi, Gn. Sibayak	KJ404924	KJ405116	KJ405295
UPOLTH0025	*Dilophotes*	AT	Sumatra, S. Utara, Brastagi, Gn. Sibayak	-	KJ405117	KJ405296
UPOLTH0026	*Dilophotes*	AT	Sumatra, S. Utara, Brastagi, Gn. Sibayak	-	KJ405118	KJ405297
UPOLTH0029	*Dilophotes*	AT	Sumatra, S. Utara, Brastagi, Gn. Sibayak	-	KJ405121	KJ405298

UPOLTH0030	*Dilophotes*	AT	Sumatra, S. Utara, Brastagi, Gn. Sibayak	-	KJ405122	KJ405299
UPOLTH0115	*Dilophotes*	AT	Sumatra, S. Utara, Brastagi, Gn. Sibayak	KJ405004	KJ405199	-
UPOLTH0116	*Dilophotes*	AT	Sumatra, S. Utara, Brastagi, Gn. Sibayak	KJ405005	KJ405200	-
UPOLTH0117	*Dilophotes*	AT	Sumatra, S. Utara, Brastagi, Gn. Sibayak	KJ405006	-	-
UPOLTH0165	*Dilophotes*	AT	Sumatra, S. Utara, Brastagi, Gn. Sibayak	KJ405053	KJ405247	KJ405373
UPOLTH0166	*Dilophotes*	AT	Sumatra, S. Utara, Brastagi, Gn. Sibayak	KJ405054	KJ405248	KJ405374
UPOLTH0169	*Dilophotes*	AT	Sumatra, S. Utara, Brastagi, Gn. Sibayak	KJ405057	KJ405251	KJ405377
UPOLTH0170	*Dilophotes*	AT	Sumatra, S. Utara, Brastagi, Gn. Sibayak	KJ405058	KJ405252	KJ405378
UPOLTH0174	*Dilophotes*	AT	Sumatra, S. Utara, Brastagi, Gn. Sibayak	KJ405062	KJ405256	KJ405382
UPOLTH0177	*Dilophotes*	AT	Sumatra, S. Utara, Brastagi, Gn. Sibayak	KJ405065	KJ405259	KJ405385
UPOLTH0178	*Dilophotes*	AT	Sumatra, S. Utara, Brastagi, Gn. Sibayak	KJ405066	KJ405260	KJ405386
UPOLTH0180	*Dilophotes*	AT	Sumatra, S. Utara, Brastagi, Gn. Sibayak	KJ405068	KJ405262	KJ405388
UPOLTH0183	*Dilophotes*	AT	Sumatra, S. Utara, Brastagi, Gn. Sibayak	KJ405071	-	KJ405391
UPOLTH0126	*Dilophotes*	AU	Philippines, Mindanao, Bagongsilang	KJ405015	KJ405209	-
UPOLTH0127	*Dilophotes*	AU	Philippines, Mindanao, Bagongsilang	KJ405016	KJ405210	KJ405357
UPOLTH0128	*Dilophotes*	AU	Philippines, Mindanao, Bagongsilang	KJ405017	KJ405211	KJ405358
UPOLTH0052	*Dilophotes*	AV	Java, S. Barat, Puncak Pass, Cipanas	KJ404943	KJ405144	-
UPOLTH0053	*Dilophotes*	AV	Java, S. Barat, Puncak Pass, Cipanas	KJ404944	KJ405145	-
UPOLTH0055	*Dilophotes*	AW	Sumatra, Jambi, Kersik Tua	KJ404946	KJ405147	-
UPOLTH0056	*Dilophotes*	AW	Sumatra, Jambi, Kersik Tua	KJ404947	KJ405148	-
UPOLTH0057	*Dilophotes*	AW	Sumatra, Jambi, Gn. Kerinci	KJ404948	KJ405149	-
UPOLTH0076	*Dilophotes*	AW	Sumatra, Jambi, Gn. Kerinci	KJ404965	KJ405164	KJ405314
UPOLTH0125	*Dilophotes*	AX	Philippines, Mindanao, Bagongsilang	KJ405014	KJ405208	KJ405356
UPOLTH0124	*Dilophotes*	AY	Philippines, Mindanao, Bagongsilang	KJ405013	KJ405207	KJ405355
UPOLTH0051	*Dilophotes*	AZ	Sumatra, Jambi, Kersik Tua, Gn. Tujuh	KJ404942	KJ405143	-
UPOLTH0035	*Dilophotes*	BA	Borneo, Sabah, Gn. Emas	KJ404927	KJ405127	-
UPOLA00001	*Dilophotes*	BB	Sumatra, S. Utara, Brastagi, Gn. Sibayak	-	KJ405079	KJ405271
UPOLTH0155	*Dilophotes*	BB	Malay Peninsula, Johor, Kotatinggi	KJ405043	KJ405237	-
UPOLTH0157	*Dilophotes*	BB	Malay Peninsula, Johor, Kotatinggi	KJ405045	KJ405239	-
UPOLTH0159	*Dilophotes*	BB	Malay Peninsula, Pahang, Tanah Rata	KJ405047	KJ405241	KJ405367

Notes. *The KJ coded Accession Numbers refer to the newly produced sequences. Other DNA sequences were taken from Bocak *et al*. (2008) and Malohlava & Bocak (2010).

**All terminals in Figs 1–4 are designated by voucher numbers.

***The multiple gene fragments are referred as *rrnL*, *cox1*, and *nad5* further.

Supplementary Table S2. Overview of colour patterns and body measurements for all analysed specimens.

Geographic origin<br>Voucher # Genus	Species	Coloration:<br>whole upper side or pronotum/elytra	Sex	Elytra Pattern Code as Fig.2	Width mm	Length mm	Detailed locality data
Asia north of Isthmus of Kra; incl. Taiwan, Hainan, and Japan							
TH0135 *Dilophotes*	A	black	female	black	0.56	5.50	Hainan, Jianfengling
TH0185 *Dilophotes*	B	black	male	black	0.65	5.15	Hua Phan, Phu Phan
TH0186 *Dilophotes*	B	black, the bases of costae 2&3 slightly lighter	female	black	0.72	5.65	Hua Phan, Phu Phan
TH0187 *Dilophotes*	B	black	male	black	0.59	4.55	Hua Phan, Phu Phan
TH0188 *Dilophotes*	B	black	male	black	0.69	5.15	Hua Phan, Phu Phan
TH0189 *Dilophotes*	B	black	male	black	0.70	5.45	Hua Phan, Phu Phan
TH0080 *Dilophotes*	E	black	female	black	0.83	6.15	Hua Phan, Ban Saluei
TH0046 *Dilophotes*	J	yellow	female	yellow	0.85	6.28	Bolikhamsai, Ban Nape
TH0078 *Dilophotes*	L	yellow	female	yellow	0.72	6.28	Hua Phan, Ban Saluei
TH0132 *Dilophotes*	N	black/reddish brown	female	red	0.95	7.05	Hua Phan, Phu Phan
TH0079 *Dilophotes*	O	dark red	female	red	0.98	7.15	Hua Phan, Ban Saluei
TH0133 *Dilophotes*	O	dark red	male	red	0.77	5.90	Hua Phan, Phu Phan
TH0134 *Dilophotes*	O	dark red	female	red	0.61	4.53	Hua Phan, Phu Phan
TH0064 *Dilophotes*	P	orange	male	yellow	0.72	4.65	Chiangmai, Doi Pui
TH0065 *Dilophotes*	P	orange	female	yellow	0.81	6.75	Chiangmai, Doi Pui
TH0070 *Dilophotes*	Q	yellow to orange	female	yellow	1.06	9.15	Arunachal, Dirang
TH0071 *Dilophotes*	Q	yellow to orange	male	yellow	0.70	6.25	Arunachal, Dirang
TH0072 *Dilophotes*	Q	yellow to orange	male	yellow	0.74	6.05	Arunachal, Dirang
TH0073 *Dilophotes*	Q	yellow to orange	female	yellow	0.73	6.60	Arunachal, Dirang
TH0102 *Dilophotes*	Q	yellow to orange	male	yellow	0.68	5.70	Arunachal, Dirang
TH0103 *Dilophotes*	Q	yellow to orange	female	yellow	0.90	7.95	Arunachal, Dirang
TH0104 *Dilophotes*	Q	yellow to orange	male	yellow	0.55	4.65	Arunachal, Dirang
TH0105 *Dilophotes*	Q	yellow to orange	male	yellow	0.74	6.25	Arunachal, Dirang
TH0106 *Dilophotes*	Q	yellow to orange	male	yellow	0.59	5.35	Arunachal, Dirang
TH0107 *Dilophotes*	Q	yellow to orange	female	yellow	0.99	8.45	Arunachal, Dirang
TH0108 *Dilophotes*	Q	yellow to orange	male	yellow	0.81	6.70	Arunachal, Dirang
TH0109 *Dilophotes*	Q	yellow to orange	female	yellow	1.02	8.25	Arunachal, Dirang

TH0110 *Dilophotes*	Q	yellow to orange	female	yellow	1.08	8.00	Arunachal, Dirang
TH0111 *Dilophotes*	Q	yellow to orange	female	yellow	0.78	6.45	Arunachal, Dirang
TH0122 *Dilophotes*	R	black/dark red	male	red	0.85	6.35	Tokushima, Tsurugisan
TH0123 *Dilophotes*	R	black/dark red	female	red	1.03	7.10	Tokushima, Akaboshiyama
TH0112 *Dilophotes*	S	black/red	male	red	0.64	5.39	Taiwan, Nantou county
TH0031 *Dilophotes*	T	dark red	male	red	0.80	6.55	Shaanxi, Qin Mts.
TH0081 *Dilophotes*	U	dark red/red	male	red	0.77	6.20	Hua Phan, Ban Saluei
TH0129 *Dilophotes*	AD	black/red, apex of elytra infuscate	female	red	0.98	5.55	Hainan, Limushan
TH0130 *Dilophotes*	AD	black/red, apex of elytra infuscate	male	red	0.62	4.40	Hainan, Limushan
TH0131 *Dilophotes*	AD	black/red, apex of elytra infuscate	female	red	0.77	5.75	Hainan, Limushan
TH0095 *Dilophotes*	AE	dark red/red	male	red	0.69	5.40	Hua Phan, Saleui
TH0190 *Dilophotes*	AF	black/dark red, with red pubescence	male	red	0.55	4.45	Guangdong, Dadong Shan
Sumatra							
TH0042 *Dilophotes*	F	yellow/yellow 1/15 infuscate	male	yellow	0.59	5.20	Pasaman, Gn. Talamau
TH0043 *Dilophotes*	F	yellow/yellow 1/15 infuscate	male	yellow	0.61	5.05	Pasaman, Gn. Talamau
TH0048 *Dilophotes*	G	yellow/yellow, apex slightly darkened	male	yellow	0.58	5.40	Brastagi, Gn. Sinnabung
A00008 *Dilophotes*	H	yellow	female	yellow	0.77	6.65	Brastagi, Gn. Sibayak
TH0007 *Dilophotes*	H	yellow	male	yellow	0.68	6.20	Brastagi, Gn. Sibayak
TH0008 *Dilophotes*	H	yellow	female	yellow	0.86	7.33	Brastagi, Gn. Sibayak
TH0009 *Dilophotes*	H	yellow	male	yellow	0.64	6.00	Brastagi, Gn. Sibayak
TH0015 *Dilophotes*	H	yellow	male	yellow	0.50	4.90	Brastagi, Gn. Sibayak
TH0041 *Dilophotes*	M	yellow/yellow slightly 1/4 infuscate	male	yellow	0.97	4.50	Pasaman, Gn. Talamau
TH0044 *Dilophotes*	M	yellow/yellow slightly 1/4 infuscate	female	yellow	0.81	6.38	Lake Maninjau, E coast
TH0049 *Dilophotes*	W	orange/orange humeri 1/4, epipleura black in apical 9/10	female	sld1	0.75	6.30	Kersik Tua, Gn. Tujuh
TH0050 *Dilophotes*	W	black/testaceous hum 2/5, epipleura black in apical 4/5	male	sld1	0.55	4.10	Kersik Tua, Gn. Tujuh
TH0074 *Dilophotes*	W	black/testaceous hum 1/2, epipleura black in apical 4/5	male	sld1	0.58	4.65	Kersik Tua, Gn. Kerinci
TH0075 *Dilophotes*	W	black/testaceous hum 3/5, epipleura black in apical 4/5	male	sld1	0.55	4.55	Kersik Tua, Gn. Kerinci
A00007 *Dilophotes*	AB	dark brown/humeri 1/8 testaceous	male	yellow & black	0.59	5.00	Brastagi, Gn. Sibayak
TH0014 *Dilophotes*	AB	dark brown/humeri 1/8 testaceous	male	yellow & black	0.56	5.05	Brastagi, Gn. Sibayak
TH0020 *Dilophotes*	AB	dark brown/humeri 1/8 testaceous	male	yellow & black	0.50	4.03	Brastagi, Gn. Sibayak
TH0021 *Dilophotes*	AB	dark brown/humeri 1/8 testaceous	male	yellow & black	0.60	4.60	Brastagi, Gn. Sibayak
TH0022 *Dilophotes*	AB	dark brown/humeri 1/8 testaceous	female	yellow & black	0.78	6.60	Brastagi, Gn. Sibayak
TH0114 *Dilophotes*	AB	dark brown/humeri 1/8 testaceous	male	yellow & black	0.55	5.00	Brastagi, Gn. Sibayak
TH0118 *Dilophotes*	AB	dark brown/humeri 1/10 testaceous	male	yellow & black	0.50	4.10	Brastagi, Gn. Sibayak

TH0120	*Dilophotes*	AB	testaceous/dark brown, humeri 1/5 testaceous	male	yellow & black	0.63	5.50	Brastagi, Gn. Sibayak
TH0121	*Dilophotes*	AB	testaceous/dark brown, humeri 1/5 testaceous	male	yellow & black	0.54	4.50	Brastagi, Gn. Sibayak
A00016	*Dilophotes*	AC	dark brown/humeral 1/4 testaceous, suture completely dark	female	yellow & humeri	0.79	6.90	Brastagi, Gn. Sibayak
TH0023	*Dilophotes*	AC	dark brown/humeral 1/4 testaceous, suture completely dark	male	yellow & humeri	0.74	6.40	Brastagi, Gn.. Sibayak
A00011	*Dilophotes*	AH	black/testaceous humeral half, epipleura black in apical 9/10	male	sld1	0.51	5.05	Brastagi, Gn. Sibayak
A00012	*Dilophotes*	AH	black/testaceous humeral half, epipleura black in apical 9/10	male	sld1	0.55	4.73	Brastagi, Gn. Sibayak
TH0006	*Dilophotes*	AH	brown/red humeral half, epipleura black in apical 3/4	female	sld1	0.68	5.65	Brastagi, Gn. Sibayak
TH0119	*Dilophotes*	AH	black/testaceous humeral half, epipleura black in apical 9/10	male	sld1	0.79	3.70	Brastagi, Gn. Sibayak
TH0175	*Dilophotes*	AH	brown/red in humeri half, epipleura black in apical 3/4	female	sld1	1.13	5.20	Brastagi, Gn. Sibayak
TH0059	*Dilophotes*	AJ	black/black, humeral 1/3 of costae testaceous	male	yellow & black	0.46	3.50	Kerinci, Muara Sako
TH0060	*Dilophotes*	AJ	black/black humeral 1/3 of costae testaceous	female	yellow & black	0.47	3.80	Kerinci, Muara Sako
A00003	*Dilophotes*	AM	brown/testaceous humeral half, epipleura black in apical 3/4	male	sld1	0.66	5.15	Brastagi, Gn. Sibayak
A00004	*Dilophotes*	AM	brown/testaceous humeral half, epipleura black in apical 4/5	male	sld1	0.50	4.35	Brastagi, Gn. Sibayak
A00006	*Dilophotes*	AM	brown/orange humeral third, epipleura black in apical 5/6	female	sld1	0.81	6.60	Brastagi, Gn. Sibayak
A00009	*Dilophotes*	AM	brown/orange humeral 2/5, epipleura black in apical 9/10	male	sld1	0.48	4.25	Brastagi, Gn. Sibayak
A00014	*Dilophotes*	AM	black/testaceous humeral 1/2, epipleura black in apical 2/3	male	sld1	0.63	3.80	Brastagi, Gn. Sibayak
TH0003	*Dilophotes*	AM	brown/orange humeral 2/5, epipleura black in apical 4/5	female	sld1	0.92	7.85	Brastagi, Gn. Sibayak
TH0010	*Dilophotes*	AM	brown/testaceous humeral 1/2, epipleura black in apical 4/5	male	sld1	0.63	5.15	Brastagi, Gn. Sibayak
TH0019	*Dilophotes*	AM	black/testaceous humeral 1/2, epipleura black in apical 4/5	male	sld1	0.52	5.00	Brastagi, Gn. Sibayak
TH0027	*Dilophotes*	AM	brown/orange humeral 2/5, epipleura black in apical 9/10	female	sld1	0.78	6.80	Brastagi, Gn. Sibayak
TH0047	*Dilophotes*	AM	black/orange humeral 2/5, epipleura black in apical 5/6	female	sld1	0.55	7.60	Brastagi, Gn. Sinnabung
TH0113	*Dilophotes*	AM	brown/orange humeral 3/5, epipleura black in apical 2/3	male	sld1	0.64	5.35	Brastagi, Gn. Sibayak
TH0167	*Dilophotes*	AM	brown/orange humeral 1/3, epipleura black in apical 9/10	female	sld1	0.56	5.45	Brastagi, Gn. Sibayak
TH0168	*Dilophotes*	AM	brown/orange humeral 1/3, epipleura black in apical 9/10	female	sld1	0.68	5.80	Brastagi, Gn. Sibayak
TH0171	*Dilophotes*	AM	brown/orange humeral 2/5, epipleura black in apical 4/5	female	sld1	0.57	5.15	Brastagi, Gn. Sibayak
TH0172	*Dilophotes*	AM	brown/orange /testaceous humeral 1/4, epipleura black	female	sld1	0.51	4.75	Brastagi, Gn. Sibayak
TH0173	*Dilophotes*	AM	brown/orange humeral 1/3, epipleura black in apical 9/10	female	sld1	0.59	5.10	Brastagi, Gn. Sibayak
TH0176	*Dilophotes*	AM	black/testaceous humeral 1/2, epipleura black in in apical 3/4	male	sld1	0.50	4.45	Brastagi, Gn. Sibayak
TH0179	*Dilophotes*	AM	brown/orange humeral 1/3, epipleura black in apical 9/10	female	sld1	0.64	5.25	Brastagi, Gn. Sibayak
TH0181	*Dilophotes*	AM	brown/orange humeral 2/5, epipleura black in apical 9/10	female	sld1	0.61	5.15	Brastagi, Gn. Sibayak
TH0182	*Dilophotes*	AM	brown/orange humeral 1/3, epipleura black in apical 9/10	female	sld1	0.58	5.15	Brastagi, Gn. Sibayak
TH0184	*Dilophotes*	AM	brown/orange humeral 1/3, epipleura black in apical 9/10	female	sld1	0.47	5.65	Brastagi, Gn. Sibayak
TH0077	*Dilophotes*	AP	black/testaceous humeral 3/4, gradual black	female	yellow & black	0.55	4.85	Kersik Tua, Gn. Kerinci
TH0083	*Dilophotes*	AP	black/testaceous humeral 1/10, apex infuscate	female	yellow & black	0.56	5.90	Gn. Merapi
TH0024	*Dilophotes*	AT	brown/orange humeral 1/2, epipleura black in apical 9/10	female	sld1	0.61	5.35	Brastagi, Gn. Sibayak

TH0025	*Dilophotes*	AT	black/orange humeral 1/2, epipleura black in apical 5/6	female	sld1	0.87	7.25	Brastagi, Gn. Sibayak
TH0026	*Dilophotes*	AT	black/orange humeral 1/2, epipleura black in apical 5/7	female	sld1	0.89	7.50	Brastagi, Gn. Sibayak
A00015	*Dilophotes*	AT	brown/testaceous humeral 1/2, 3/4 of epipleura black	male	sld1	0.52	4.05	Brastagi, Gn. Sibayak
TH0002	*Dilophotes*	AT	dark brown/humeral 1/2 testaceous, epipleura compl. black	male	sld1	0.54	4.50	Brastagi, Gn. Sibayak
A00010	*Dilophotes*	AT	brown/testaceous humeral 1/2, 2/3 of epipleura black	male	sld1	0.52	4.40	Brastagi, Gn. Sibayak,
TH0018	*Dilophotes*	AT	testaceous/humeral 3/5 testaceous, 2/3 of epipleura black	male	sld1	0.56	4.50	Brastagi, Gn. Sibayak
A00002	*Dilophotes*	AT	black/humeral 1/2 orange, epipleura black in apical 3/4	female	sld1	0.80	6.80	Brastagi, Gn. Sibayak
TH0001	*Dilophotes*	AT	black/humeral 1/2 orange, epipleura black in apical 9/10	female	sld1	0.77	6.05	Brastagi, Gn. Sibayak
A00005	*Dilophotes*	AT	black/humeral 1/2 orange, epipleura black in apical 9/11	female	sld1	0.74	6.00	Brastagi, Gn. Sibayak
TH0016	*Dilophotes*	AT	brown/testaceous humeral 1/2, epipleura black in apical 3/4	male	sld1	0.57	4.75	Brastagi, Gn. Sibayak
TH0004	*Dilophotes*	AT	dark testaceous/humeral 2/3 orange, epipl. black in apical 3/4	male	sld1	0.53	5.35	Brastagi, Gn. Sibayak
TH0005	*Dilophotes*	AT	brown/orange humeral 1/2, epipleura black in apical 9/10	female	sld1	0.69	5.80	Brastagi, Gn. Sibayak
TH0012	*Dilophotes*	AT	brown/testaceous humeral 3/5, epipleura black in apical 2/3	male	sld1	0.57	5.00	Brastagi, Gn. Sibayak
TH0013	*Dilophotes*	AT	brown/testaceous humeral 1/2, epipleura black in apical 3/4	male	sld1	0.55	4.65	Brastagi, Gn. Sibayak
TH0028	*Dilophotes*	AT	brown/orange humeral 1/2, epipleura black in apical 2/3	female	sld1	0.89	7.50	Brastagi, Gn. Sibayak
TH0029	*Dilophotes*	AT	black/humeral 1/2 orange, epipleura black in apical 9/10	female	sld1	0.69	6.25	Brastagi, Gn. Sibayak
TH0030	*Dilophotes*	AT	black/humeral 1/2 orange, epipleura black in apical 9/11	female	sld1	0.72	6.30	Brastagi, Gn. Sibayak
TH0115	*Dilophotes*	AT	testaceous/humeral 3/5 testaceous, 2/3 of epipleura black	male	sld1	0.59	5.25	Brastagi, Gn. Sibayak
TH0116	*Dilophotes*	AT	black/testaceous humeral 1/2, epipl. black in apical 3/4 length	male	sld1	0.56	4.75	Brastagi, Gn. Sibayak
TH0117	*Dilophotes*	AT	brown/testaceous humeral 3/5, epipleura black apical 3/4	male	sld1	0.61	5.05	Brastagi, Gn. Sibayak
TH0165	*Dilophotes*	AT	brown/testaceous humeral 1/2, epipleura black in apical 3/4	male	sld1	0.54	4.75	Brastagi, Gn. Sibayak
TH0166	*Dilophotes*	AT	black/testaceous humeral 1/2, epipleura black in apical 9/10	female	sld1	0.55	6.15	Brastagi, Gn. Sibayak
TH0169	*Dilophotes*	AT	black/testaceous humeral 1/2, epipleura black in apical 4/5	male	sld1	0.54	4.75	Brastagi, Gn. Sibayak
TH0170	*Dilophotes*	AT	black/orange humeral 1/2, epipleura black in apical 4/	female	sld1	0.68	5.65	Brastagi, Gn. Sibayak
TH0174	*Dilophotes*	AT	black/orange humeral 1/2, epipleura completely black	female	sld1	0.59	5.55	Brastagi, Gn. Sibayak
TH0177	*Dilophotes*	AT	black/testaceous humeral 1/2, epipleura black in apical 9/10	female	sld1	0.64	5.40	Brastagi, Gn. Sibayak
TH0178	*Dilophotes*	AT	brown/orange humeral 1/2, epipleura black in apical 9/10	female	sld1	0.64	5.90	Brastagi, Gn. Sibayak
TH0180	*Dilophotes*	AT	brown/orange humeral 1/2, epipleura black in apical 4/5	female	sld1	0.61	5.10	Brastagi, Gn. Sibayak
TH0183	*Dilophotes*	AT	black/testaceous humeral 1/2, epipleura black in apical 9/10	male	sld1	0.58	5.15	Brastagi, Gn. Sibayak
TH0055	*Dilophotes*	AW	black/orange humeral 5/6, epipleura same color	female	sld1	0.77	6.25	Kersik Tua
TH0056	*Dilophotes*	AW	black/orange, apex slightly infuscate	female	sld1	0.87	7.25	Kersik Tua
TH0057	*Dilophotes*	AW	black/testaceous humeral 3/5, epipleura same color	male	sld1	0.55	4.65	Gn. Kerinci
TH0076	*Dilophotes*	AW	black/testaceous humeral 3/5, epipleura same color	male	sld1	0.51	4.20	Gn. Kerinci
TH0051	*Dilophotes*	AZ	brown/orange humeral 1/2, epipleura same color	female	red & black*	0.59	5.05	Kersik Tua, Gn. Tujuh
A00001	*Dilophotes*	BB	dark brown, humeral 2/3 orange, epipleura completely black	female	red & black*	0.58	6.75	Brastagi, Gn. Sibayak

Malay Peninsula							
TH0139 *Dilophotes*	D	black/ costae in humeral 1/5 testaceous	male	striped	0.66	4.65	Johor, Kotatinggi
TH0141 *Dilophotes*	D	black/ costae in humeral 1/5 testaceous	female	striped	0.76	5.15	Kelantan, Gua Musang
TH0143 *Dilophotes*	D	black/ costae in humeral 1/5 testaceous	male	striped	0.56	4.60	Kelantan, Gua Musang
TH0144 *Dilophotes*	D	black/ costae in humeral 1/5 testaceous	male	striped	0.63	4.80	Kelantan, Gua Musang
TH0154 *Dilophotes*	C	black	male	black	0.70	6.20	Johor, Kotatinggi
TH0061 *Dilophotes*	I	black	male	black	0.57	5.85	Pahang, Road Ipoh Kg. Raja
TH0063 *Dilophotes*	I	black	female	black	0.78	6.40	Pahang, Tanah Rata
TH0038 *Dilophotes*	K	black	male	black	0.72	6.15	Pahang, Gn. Jasar
TH0068 *Dilophotes*	K	black	male	black	0.55	5.00	Pahang, Tanah Rata
TH0147 *Dilophotes*	K	black	male	black	0.69	6.30	Kelantan, Lal. Pandrak
TH0156 *Dilophotes*	K	black	female	black	0.95	7.88	Johor, Kotatinggi
TH0136 *Dilophotes*	K	black	male	black	0.88	7.05	Pahang, Gn. Jasar
TH0151 *Dilophotes*	K	black	female	black	0.56	5.45	Kelantan, Lal. Pandrak
TH0153 *Dilophotes*	K	black	male	black	0.58	5.15	Johor, Kotatinggi
TH0148 *Dilophotes*	Y	black	male	black	0.72	5.95	Kelantan, Gua Musang
TH0152 *Dilophotes*	Y	black	female	black	0.99	8.25	Pahang, Gn. Berembun
TH0158 *Dilophotes*	Y	black	male	black	0.74	6.45	Johor, Kotatinggi
TH0164 *Dilophotes*	Y	black	male	black	0.69	5.95	Kelantan, Lal. Pandrak
TH0160 *Dilophotes*	Z	black	male	black	0.76	5.95	Kelantan, Lal. Pandrak
TH0069 *Dilophotes*	AL	black, only costae 1&2 testaceous in humeral third	male	striped	0.47	5.20	Pahang, Tanah Rata
TH0137 *Dilophotes*	AM	black, costae 1&2 testaceous till middle, costa 3 basally	male	striped	0.57	4.25	Johor, Kotatinggi
TH0140 *Dilophotes*	AM	black, costae 1&2 testaceous till middle, costa 3 basally	male	striped	0.58	4.40	Kelantan, Gua Musang
TH0142 *Dilophotes*	AM	black, humeri 3/5 brown, costae testaceous, apical 2/5 dark	female	striped	0.88	6.50	Kelantan, Gua Musang
TH0145 *Dilophotes*	AM	costae 1&2 testaceous till middle, costa 3 basally	male	striped	0.55	3.90	Kelantan, Gua Musang
TH0146 *Dilophotes*	AO	black	male	black	0.57	6.15	Kelantan, L. Pandrak
TH0161 *Dilophotes*	AO	black	male	black	0.61	4.90	Kelantan, L. Pandrak
TH0162 *Dilophotes*	AO	black	male	black	0.57	4.75	Kelantan, L. Pandrak
TH0163 *Dilophotes*	AO	black	female	black	0.91	7.30	Kelantan, Lal. Pandrak
TH0138 *Dilophotes*	AR	black	male	black	0.44	3.33	Johor, Kotatinggi
TH0155 *Dilophotes*	BB	black	male	black	0.54	4.30	Johor, Kotatinggi
TH0157 *Dilophotes*	BB	black	male	black	0.54	5.08	Johor, Kotatinggi
TH0159 *Dilophotes*	BB	black	female	black	0.99	7.90	Pahang, Tanah Rata

Borneo								
TH0037	*Dilophotes*	V	black/red humeral 1/3, non gradual, epipleura same colour	female	red & black*	1.01	8.15	Sabah, Gn. Emas
000244	*Dilophotes*	AA	black/testaceous humeral 1/3, epipleura same color	female	yellow &black	1.35	5.15	Sabah, Gn. Emas
TH0093	*Dilophotes*	AG	black/testaceous humeral 1/3, epipleura black in whole length	male	yellow &black*	0.56	4.28	Kalim. Selatan, Loksado
TH0101	*Dilophotes*	AI	black/brown humeral 1/2, epipleura black	male	sld1	0.47	3.55	Kalim. Selatan, Loksado
TH0088	*Dilophotes*	AI	black/ humeral 1/3 light	male	sld1	0.55	4.80	Kalim. Selatan, Loksado
TH0091	*Dilophotes*	AI	black/brown humeral 1/2, epipleura completely black	male	sld1	0.62	4.10	Kalim. Selatan, Loksado
TH0096	*Dilophotes*	AI	black/humeri slightly lighter	male	sld1	0.62	4.60	Kalim. Selatan, Loksado
TH0097	*Dilophotes*	AI	black, 1/2 dark orange, costae lighter, epipleura black	female	sld1	0.47	5.85	Kalim. Selatan, Loksado
TH0099	*Dilophotes*	AI	black/brown humeral 1/2, epipleura completely black	male	sld1	0.56	4.40	Kalim. Selatan, Loksado
TH0100	*Dilophotes*	AI	black/humeri slightly lighter	male	sld1	0.54	4.15	Kalim. Selatan, Loksado
TH0066	*Dilophotes*	AI	black/orange humeral 1/2, epipleura black in apical 3/4	female	sld1	0.64	5.15	Sabah, Gn. Emas
TH0032	*Dilophotes*	AQ	black/orange, apical 2/5 black	male	red & black	0.64	5.75	Sabah, Gn. Emas
TH0034	*Dilophotes*	AQ	black/orange, apical 2/3 black	male	red & black	0.64	5.30	Sabah, Gn. Emas
TH0036	*Dilophotes*	AQ	black/orange, apical 3/5 black	female	red & black	0.66	5.85	Sabah, Gn. Emas
TH0067	*Dilophotes*	AQ	black/orange, apical 2/5 black	male	red & black	0.67	0.70	Sabah, Gn. Emas
TH0033	*Dilophotes*	AS	black	male	sld2	0.52	4.15	Kalim. Tenggah, M. Teweh
TH0089	*Dilophotes*	AS	black	male	sld2	0.58	4.30	Kalim. Selatan, Loksado
TH0090	*Dilophotes*	AS	black/brown humeral 1/3, costae lighter, epipleura black,	female	sld2	0.65	5.10	Kalim. Selatan, Loksado
TH0092	*Dilophotes*	AS	black/brown humeral 1/3, costae lighter, epipleura black,	female	sld2	0.68	5.90	Kalim. Selatan, Loksado
TH0098	*Dilophotes*	AS	black/brown humeral 1/3, costae lighter, epipleura black,	female	sld2	0.67	5.35	Kalim. Selatan, Loksado
TH0035	*Dilophotes*	BA	black/orange humeral 3/5, epipleura same color	female	red & black*	0.72	5.85	Sabah, Gn. Emas
Java								
TH0039	*Dilophotes*	AK	brown/orange humeral 2/3, epipleura same color	female	red & black*	0.83	6.80	Java Tenggah, Gn. Lawu
TH0040	*Dilophotes*	AK	black/orange humeral 2/3, epipleura same color	female	red & black*	0.90	7.95	Java Tenggah, Gn. Lawu
TH0054	*Dilophotes*	AN	black/orange, apical 1/10 darkened	female	red & black*	0.77	6.35	Puncak Pass, Cipanas
TH0052	*Dilophotes*	AV	orange/orange humeral 3/5, epipleura same color	female	sld1	0.79	7.10	Puncak Pass, Cipanas
TH0053	*Dilophotes*	AV	yellow/yellow humeral 2/3, epipleura same color	male	sld1	0.61	5.50	Puncak Pass, Cipanas

Philippines							
A00060 *Dilophotes*	X	black	female	black	0.80	6.65	Negros, Mt. Canlaon
TH0126 *Dilophotes*	AU	black/testaceous humeral 1/4, epipleura black	male	yellow & black	0.57	4.20	Mindanao, Bagongsilang
TH0127 *Dilophotes*	AU	black/testaceous humeral 1/4, epipleura black	male	yellow & black	0.58	4.75	Mindanao, Bagongsilang
TH0128 *Dilophotes*	AU	black/testaceous humeral 1/3, epipleura black in apical 9/10	female	yellow & black	0.65	5.35	Mindanao, Bagongsilang
TH0124 *Dilophotes*	AY	orange/orange humeral 1/2, epipleura same color	female	red & black*	0.63	5.05	Mindanao, Bagongsilang
TH0125 *Dilophotes*	AX	black/ brownish pubescent	female	black	0.61	5.20	Mindanao, Bagongsilang

*If only a single sex is available and resembles a pattern present in monomorphic and dimorphic species, the species was coded as monomorphic.

Supplementary Table S3. The list of sequenced individuals and delineation of biological species based on morphology and GMYC analyses.

Voucher Number	Genus	Species identifications consensus of the GMYC analyses and morphology	GMYC analysis based on mtDNA fragment: *rrnL*	*cox1*	*nad5*	Geographic origin
UPOLTH0135	*Dilophotes*	A	A	A	-	China, Hainan, Jianfengling
UPOLTH0185	*Dilophotes*	B	B	B	B	Laos, Hua Phan, Phu Phan
UPOLTH0186	*Dilophotes*	B	B	B	B	Laos, Hua Phan, Phu Phan
UPOLTH0187	*Dilophotes*	B	B	-	B	Laos, Hua Phan, Phu Phan
UPOLTH0188	*Dilophotes*	B	B	B	B	Laos, Hua Phan, Phu Phan
UPOLTH0189	*Dilophotes*	B	B	B	B	Laos, Hua Phan, Phu Phan
UPOLTH0154	*Dilophotes*	C	C	C	-	Malay Peninsula, Johor, Kotatinggi
UPOLTH0139	*Dilophotes*	D	Da	Da	Da	Malay Peninsula, Johor, Kotatinggi
UPOLTH0141	*Dilophotes*	D	Db	Db	-	Malay Peninsula, Kelantan, Gua Musang
UPOLTH0143	*Dilophotes*	D	Db	Db	Db	Malay Peninsula, Kelantan, Gua Musang
UPOLTH0144	*Dilophotes*	D	Db	Db	Db	Malay Peninsula, Kelantan, Gua Musang
UPOLTH0080	*Dilophotes*	E	E	E	E	Laos, Hua Phan prov., Ban Saluei
UPOLTH0042	*Dilophotes*	F	-	F	-	Sumatra, Barat, Pasaman, Gn. Talamau
UPOLTH0043	*Dilophotes*	F	F	F	-	Sumatra, Barat, Pasaman, Gn. Talamau
UPOLTH0048	*Dilophotes*	G	G	G	G	Sumatra, Utara, Brastagi, Gn. Sinnabung
UPOLA00008	*Dilophotes*	H	Ha	H	H	Sumatra, Utara, Brastagi, Gn. Sibayak
UPOLTH0007	*Dilophotes*	H	Ha	H	H	Sumatra, Utara, Brastagi, Gn. Sibayak
UPOLTH0008	*Dilophotes*	H	Ha	H	H	Sumatra, Utara, Brastagi, Gn. Sibayak
UPOLTH0009	*Dilophotes*	H	Hb	H	H	Sumatra, Utara, Brastagi, Gn. Sibayak
UPOLTH0015	*Dilophotes*	H	Ha	H	-	Sumatra, Utara, Brastagi, Gn. Sibayak
UPOLTH0061	*Dilophotes*	I	I	-	I	Malay Peninsula, Pahang, Rd Ipoh-Kg. Raja
UPOLTH0063	*Dilophotes*	I	I	-	I	Malay Peninsula, Pahang, Tanah Rata
UPOLTH0046	*Dilophotes*	J	J	J	-	Laos, Bolikhamsai pr., Ban Nape
UPOLTH0038	*Dilophotes*	K	K	K	-	Malay Peninsula, Pahang, Gn. Jasar
UPOLTH0068	*Dilophotes*	K	K	K	K	Malay Peninsula, Pahang, Tanah Rata
UPOLTH0136	*Dilophotes*	K	K	K	-	Malay Peninsula, Pahang, Gn. Jasar
UPOLTH0147	*Dilophotes*	K	-	K	-	Malay Peninsula, Kelantan, Ladang Pandrak
UPOLTH0151	*Dilophotes*	K	K	K	-	Malay Peninsula, Kelantan, Ladang Pandrak
UPOLTH0153	*Dilophotes*	K	K	K	-	Malay Peninsula, Johor, Kota Tinggi
UPOLTH0156	*Dilophotes*	K	K	K	K	Malay Peninsula, Johor, Kota Tinggi
UPOLTH0078	*Dilophotes*	L	L	L	L	Laos, Hua Phan prov., Ban Saluei
UPOLTH0041	*Dilophotes*	M	Ma	Ma	-	Sumatra, Barat, Pasaman, Gn. Talamau

UPOLTH0044	*Dilophotes*	M	Mb	Mb	-	Sumatra, Barat, Lake Maninjau
UPOLTH0132	*Dilophotes*	N	N	N	N	Laos, Hua Phan, Phu Phan
UPOLTH0079	*Dilophotes*	O	O	O	O	Laos, Hua Phan prov., Ban Saluei
UPOLTH0133	*Dilophotes*	O	O	O	-	Laos, Hua Phan prov., Phu Phan
UPOLTH0134	*Dilophotes*	O	O	O	-	Laos, Hua Phan prov., Phu Phan
UPOLTH0064	*Dilophotes*	P	P	-	-	Thailand, Chiangmai pr., Doi Pui
UPOLTH0065	*Dilophotes*	P	P	P	-	Thailand, Chiangmai pr., Doi Pui
UPOLTH0070	*Dilophotes*	Q	Q	Q	Q	India, Arunachal, Dirang
UPOLTH0071	*Dilophotes*	Q	Q	Q	Q	India, Arunachal, Dirang
UPOLTH0072	*Dilophotes*	Q	Q	Q	Q	India, Arunachal, Dirang
UPOLTH0073	*Dilophotes*	Q	Q	Q	Q	India, Arunachal, Dirang
UPOLTH0102	*Dilophotes*	Q	Q	Q	Q	India, Arunachal, Dirang
UPOLTH0103	*Dilophotes*	Q	Q	Q	Q	India, Arunachal, Dirang
UPOLTH0104	*Dilophotes*	Q	Q	Q	Q	India, Arunachal, Dirang
UPOLTH0105	*Dilophotes*	Q	Q	Q	Q	India, Arunachal, Dirang
UPOLTH0106	*Dilophotes*	Q	Q	Q	Q	India, Arunachal, Dirang
UPOLTH0107	*Dilophotes*	Q	Q	Q	Q	India, Arunachal, Dirang
UPOLTH0108	*Dilophotes*	Q	Q	Q	Q	India, Arunachal, Dirang
UPOLTH0109	*Dilophotes*	Q	Q	Q	Q	India, Arunachal, Dirang
UPOLTH0110	*Dilophotes*	Q	Q	Q	Q	India, Arunachal, Dirang
UPOLTH0111	*Dilophotes*	Q	Q	Q	Q	India, Arunachal, Dirang
UPOLTH0122	*Dilophotes*	R	R	Ra	Ra	Japan, Tokushima, Tsurugisan
UPOLTH0123	*Dilophotes*	R	R	Rb	Rb	Japan, Tokushima, Akaboshiyama
UPOLTH0112	*Dilophotes*	S	S	S	S	China, Taiwan, Nantou county
UPOLTH0031	*Dilophotes*	T	T	T	-	China, Shaanxi, Qin Mts.
UPOLTH0081	*Dilophotes*	U	U	U	U	Laos, Hua Phan prov., Ban Saluei
UPOLTH0037	*Dilophotes*	V	VI	VII	-	Borneo, Sabah, Gn. Emas
UPOLTH0049	*Dilophotes*	W	W	W	-	Sumatra, Jambi, Kersik Tua, Gn. Tujuh
UPOLTH0050	*Dilophotes*	W	W	W	-	Sumatra, Jambi, Kersik Tua, Gn. Tujuh
UPOLTH0074	*Dilophotes*	W	W	W	W	Sumatra, Jambi, Kersik Tua, Gn. Kerinci
UPOLTH0075	*Dilophotes*	W	W	W	W	Sumatra, Jambi, Kersik Tua, Gn. Kerinci
UPOLA00060	*Dilophotes*	X	XI	XII	I	Philippines, Negros, Mt. Canlaon
UPOLTH0148	*Dilophotes*	Y	Y	Y	-	Malay Pen., Kelantan, Ladang Pandrak
UPOLTH0152	*Dilophotes*	Y	Y	Y	Y	Malay Pen., Pahang, Gn. Beremban
UPOLTH0158	*Dilophotes*	Y	Y	Y	-	Malay Peninsula, Johor, Kota Tinggi
UPOLTH0164	*Dilophotes*	Y	Y	Y	Y	Malay Peninsula, Kelantan, Ladang Pandrak
UPOLTH0160	*Dilophotes*	Z	Z	Z	Z	Malay Peninsula, Kelantan, Ladang Pandrak

UPOL000244	*Dilophotes*	AA	AA	AA	AA	Borneo, Sabah, Gn. Emas
UPOLA00007	*Dilophotes*	AB	ABa	AB	-	Sumatra, Utara, Brastagi, Gn. Sibayak
UPOLTH0014	*Dilophotes*	AB	Abb	AB	-	Sumatra, Utara, Brastagi, Gn. Sibayak
UPOLTH0020	*Dilophotes*	AB	ABa	AB	AB	Sumatra, Utara, Brastagi, Gn. Sibayak
UPOLTH0021	*Dilophotes*	AB	ABa	AB	AB	Sumatra, Utara, Brastagi, Gn. Sibayak
UPOLTH0022	*Dilophotes*	AB	ABa	AB	-	Sumatra, Utara, Brastagi, Gn. Sibayak
UPOLTH0114	*Dilophotes*	AB	ABa	AB	AB	Sumatra, Utara, Brastagi, Gn. Sibayak
UPOLTH0118	*Dilophotes*	AB	ABa	AB	-	Sumatra, Utara, Brastagi, Gn. Sibayak
UPOLTH0120	*Dilophotes*	AB	ABa	AB	-	Sumatra, Utara, Brastagi, Gn. Sibayak
UPOLTH0121	*Dilophotes*	AB	ABa	AB	-	Sumatra, Utara, Brastagi, Gn. Sibayak
UPOLA00016	*Dilophotes*	AC	AC	AC	-	Sumatra, Utara, Brastagi, Gn. Sibayak
UPOLTH0023	*Dilophotes*	AC	AC!	AC	AB	Sumatra, Utara, Brastagi, Gn.. Sibayak
UPOLTH0129	*Dilophotes*	AD	AD	ADa	-	China, Hainan, Limushan
UPOLTH0130	*Dilophotes*	AD	AD	ADa	AD	China, Hainan, Limushan
UPOLTH0131	*Dilophotes*	AD	AD	ADb	-	China, Hainan, Limushan
UPOLTH0095	*Dilophotes*	AE	AE	AE	AE	Laos, Hua Phan, Saleui
UPOLTH0190	*Dilophotes*	AF	AF	AF	AF	China, Guangdong, Dadong Shan
UPOLTH0093	*Dilophotes*	AG	AG	AG	AG	Borneo, Kalimantan Selatan, Loksado
UPOLA00011	*Dilophotes*	AH	AH	AH	-	Sumatra, Utara, Brastagi, Gn. Sibayak
UPOLA00012	*Dilophotes*	AH	AH	AH	-	Sumatra, Utara, Brastagi, Gn. Sibayak
UPOLTH0006	*Dilophotes*	AH	AH	AH	AH	Sumatra, Utara, Brastagi, Gn. Sibayak
UPOLTH0119	*Dilophotes*	AH	AH	AH	AH	Sumatra, Utara, Brastagi, Gn. Sibayak
UPOLTH0175	*Dilophotes*	AH	AH	AH	AH	Sumatra, Utara, Brastagi, Gn. Sibayak
UPOLTH0088	*Dilophotes*	AI	AIa	AIa	-	Borneo, Kalimantan Selatan, Loksado
UPOLTH0091	*Dilophotes*	AI	AIa	AIa	AIa	Borneo, Kalimantan Selatan, Loksado
UPOLTH0096	*Dilophotes*	AI	AIa	AIa	AIa	Borneo, Kalimantan Selatan, Loksado
UPOLTH0097	*Dilophotes*	AI	AIa	AIa	AIa	Borneo, Kalimantan Selatan, Loksado
UPOLTH0099	*Dilophotes*	AI	AIa	AIa	AIa	Borneo, Kalimantan Selatan, Loksado
UPOLTH0100	*Dilophotes*	AI	AIa	AIa	AIa	Borneo, Kalimantan Selatan, Loksado
UPOLTH0101	*Dilophotes*	AI	AIa	AIa	AIa	Borneo, Kalimantan Selatan, Loksado
UPOLTH0066	*Dilophotes*	AI	AIb	AIb	-	Borneo, Sabah, Gn. Emas
UPOLTH0059	*Dilophotes*	AJ	AJ	AJ	-	Sumatra, Barat, Muara Sako
UPOLTH0060	*Dilophotes*	AJ	-	AJ	-	Sumatra, Barat, Muara Sako
UPOLTH0039	*Dilophotes*	AK	AK	AK	-	Java, Tenggah, Gn. Lawu, Sarangan
UPOLTH0040	*Dilophotes*	AK	AK	AK	-	Java, Tenggah, Gn. Lawu, Sarangan
UPOLTH0069	*Dilophotes*	AL	AL	AL	-	Malay Peninsula, Pahang, Tanah Rata
UPOLA00003	*Dilophotes*	AM	AMa	AMa	AMa	Sumatra, Utara, Brastagi, Gn. Sibayak

UPOLA00004	*Dilophotes*	AM	AMa	AMa	AMa	Sumatra, Utara, Brastagi, Gn. Sibayak
UPOLA00006	*Dilophotes*	AM	AMa	AMa	AMa	Sumatra, Utara, Brastagi, Gn. Sibayak
UPOLA00009	*Dilophotes*	AM	AMa	AMa	AMa	Sumatra, Utara, Brastagi, Gn. Sibayak
UPOLA00014	*Dilophotes*	AM	AMa	AMa	AMa	Sumatra, Utara, Brastagi, Gn. Sibayak
UPOLTH0003	*Dilophotes*	AM	AMa	AMa	AMa	Sumatra, Utara, Brastagi, Gn. Sibayak
UPOLTH0010	*Dilophotes*	AM	AMa	AMa	AMa	Sumatra, Utara, Brastagi, Gn. Sibayak
UPOLTH0011	*Dilophotes*	AM	AMa	-	-	Sumatra, Utara, Brastagi, Gn. Sibayak
UPOLTH0017	*Dilophotes*	AM	AMa	AMa	-	Sumatra Utara, Brastagi, Gn. Sibayak
UPOLTH0019	*Dilophotes*	AM	AMa	AMa	AMa	Sumatra, Utara, Brastagi, Gn. Sibayak
UPOLTH0027	*Dilophotes*	AM	-	AMa	-	Sumatra, Utara, Brastagi, Gn. Sibayak
UPOLTH0028	*Dilophotes*	AM	-	AMa	-	Sumatra, Utara, Brastagi, Gn. Sibayak
UPOLTH0047	*Dilophotes*	AM	AMa	AMa	AMa	Sumatra, Utara, Brastagi, Gn. Sinnabung
UPOLTH0113	*Dilophotes*	AM	AMa	AMa	-	Sumatra, Utara, Brastagi, Gn. Sibayak
UPOLTH0167	*Dilophotes*	AM	AMa	AMa	AMa	Sumatra, Utara, Brastagi, Gn. Sibayak
UPOLTH0168	*Dilophotes*	AM	AMa	AMa	AMa	Sumatra, Utara, Brastagi, Gn. Sibayak
UPOLTH0171	*Dilophotes*	AM	AMa	AMa	AMa	Sumatra, Utara, Brastagi, Gn. Sibayak
UPOLTH0172	*Dilophotes*	AM	AMa	AMa	AMa	Sumatra, Utara, Brastagi, Gn. Sibayak
UPOLTH0173	*Dilophotes*	AM	AMa	AMa	AMa	Sumatra, Utara, Brastagi, Gn. Sibayak
UPOLTH0176	*Dilophotes*	AM	AMa	AMa	AMa	Sumatra, Utara, Brastagi, Gn. Sibayak
UPOLTH0179	*Dilophotes*	AM	AMa	AMa	AMa	Sumatra, Utara, Brastagi, Gn. Sibayak
UPOLTH0181	*Dilophotes*	AM	AMa	AMa	AMa	Sumatra, Utara, Brastagi, Gn. Sibayak
UPOLTH0182	*Dilophotes*	AM	AMa	AMa	AMa	Sumatra, Utara, Brastagi, Gn. Sibayak
UPOLTH0184	*Dilophotes*	AM	AMa	AMa	AMa	Sumatra Utara, Brastagi, Gn. Sibayak
UPOLTH0140	*Dilophotes*	AM	AMb	AMb	-	Malay Peninsula, Kelantan, Gua Musang
UPOLTH0142	*Dilophotes*	AM	AMb	AMb	-	Malay Peninsula, Kelantan, Gua Musang
UPOLTH0145	*Dilophotes*	AM	AMb	AMb	-	Malay Peninsula, Kelantan, Gua Musang
UPOLTH0137	*Dilophotes*	AM	AMc	AMc	-	Malay Peninsula, Johor, Kotatinggi
UPOLTH0054	*Dilophotes*	AN	AN	AN	-	Java, Barat, Puncak Pass, Cipanas
UPOLTH0146	*Dilophotes*	AO	AO	AO	-	Malay Peninsula, Kelantan, Ladang Pandrak
UPOLTH0161	*Dilophotes*	AO	AO	AO	AO	Malay Peninsula, Kelantan, Ladang Pandrak
UPOLTH0162	*Dilophotes*	AO	AO	AO	AO	Malay Peninsula, Kelantan, Ladang Pandrak
UPOLTH0163	*Dilophotes*	AO	AO	AO	AO	Malay Peninsula, Kelantan, Ladang Pandrak
UPOLTH0077	*Dilophotes*	AP	AP	APa	APa	Sumatra, Jambi, Kersik Tua, Gn. Kerinci
UPOLTH0083	*Dilophotes*	AP	AP	APb	APb	Sumatra, Barat, Gn. Merapi
UPOLTH0032	*Dilophotes*	AQ	-	AQa	-	Borneo, Sabah, Gn. Emas
UPOLTH0034	*Dilophotes*	AQ	-	AQa	-	Borneo, Sabah, Gn. Emas
UPOLTH0036	*Dilophotes*	AQ	AQb	AQb	-	Borneo, Sabah, Gn. Emas
UPOLTH0067	*Dilophotes*	AQ	AQa	AQa	AQa	Borneo, Sabah, Gn. Emas
UPOLTH0138	*Dilophotes*	AR	AR	AR	AR	Malay Peninsula, Johor, Kotatinggi

UPOLTH0033	*Dilophotes*	AS	ASa	AS	-	Borneo, Kalim. Tenggah, Muara Teweh
UPOLTH0089	*Dilophotes*	AS	ASb	AS	AS	Borneo, Kalimantan Selatan, Loksado
UPOLTH0090	*Dilophotes*	AS	ASb	AS	AS	Borneo, Kalimantan Selatan, Loksado
UPOLTH0092	*Dilophotes*	AS	ASb	AS	AS	Borneo, Kalimantan Selatan, Loksado
UPOLTH0098	*Dilophotes*	AS	ASb	AS	AS	Borneo, Kalimantan Selatan, Loksado
UPOLA00002	*Dilophotes*	AT	AT	AT	-	Sumatra, Utara, Brastagi, Gn. Sibayak
UPOLA00005	*Dilophotes*	AT	AT	AT	-	Sumatra, Utara, Brastagi, Gn. Sibayak
UPOLA00010	*Dilophotes*	AT	AT	AT	-	Sumatra, Utara, Brastagi, Gn. Sibayak,
UPOLA00013	*Dilophotes*	AT	AT	AT	-	Sumatra, Utara, Brastagi, Gn. Sibayak
UPOLA00015	*Dilophotes*	AT	AT	AT	-	Sumatra, Utara, Brastagi, Gn. Sibayak
UPOLTH0001	*Dilophotes*	AT	AT	AT	AT	Sumatra, Utara, Brastagi, Gn. Sibayak
UPOLTH0002	*Dilophotes*	AT	AT	AT	AT	Sumatra, Utara, Brastagi, Gn. Sibayak
UPOLTH0004	*Dilophotes*	AT	AT	AT	AT	Sumatra, Utara, Brastagi, Gn. Sibayak
UPOLTH0005	*Dilophotes*	AT	AT	AT	AT	Sumatra, Utara, Brastagi, Gn. Sibayak
UPOLTH0012	*Dilophotes*	AT	AT	AT	AT	Sumatra, Utara, Brastagi, Gn. Sibayak
UPOLTH0016	*Dilophotes*	AT	AT	AT	AT	Sumatra, Utara, Brastagi, Gn. Sibayak
UPOLTH0018	*Dilophotes*	AT	AT	AT	AT	Sumatra, Utara, Brastagi, Gn. Sibayak
UPOLTH0024	*Dilophotes*	AT	AT	AT	AT	Sumatra, Utara, Brastagi, Gn. Sibayak
UPOLTH0025	*Dilophotes*	AT	-	AT	AE	Sumatra, Utara, Brastagi, Gn. Sibayak
UPOLTH0026	*Dilophotes*	AT	-	AT	AE	Sumatra, Utara, Brastagi, Gn. Sibayak
UPOLTH0029	*Dilophotes*	AT	-	AT	AE	Sumatra, Utara, Brastagi, Gn. Sibayak
UPOLTH0030	*Dilophotes*	AT	-	AT	AE	Sumatra, Utara, Brastagi, Gn. Sibayak
UPOLTH0115	*Dilophotes*	AT	AT	AT	-	Sumatra, Utara, Brastagi, Gn. Sibayak
UPOLTH0116	*Dilophotes*	AT	AT	AT	-	Sumatra, Utara, Brastagi, Gn. Sibayak
UPOLTH0117	*Dilophotes*	AT	AT	-	-	Sumatra, Utara, Brastagi, Gn. Sibayak
UPOLTH0165	*Dilophotes*	AT	AT	AT	AT	Sumatra, Utara, Brastagi, Gn. Sibayak
UPOLTH0166	*Dilophotes*	AT	AT	AT	AT	Sumatra, Utara, Brastagi, Gn. Sibayak
UPOLTH0169	*Dilophotes*	AT	AT	AT	AT	Sumatra, Utara, Brastagi, Gn. Sibayak
UPOLTH0170	*Dilophotes*	AT	AT	AT	AT	Sumatra, Utara, Brastagi, Gn. Sibayak
UPOLTH0174	*Dilophotes*	AT	AT	AT	AT	Sumatra, Utara, Brastagi, Gn. Sibayak
UPOLTH0177	*Dilophotes*	AT	AT	AT	AT	Sumatra, Utara, Brastagi, Gn. Sibayak
UPOLTH0178	*Dilophotes*	AT	AT	AT	AT	Sumatra, Utara, Brastagi, Gn. Sibayak
UPOLTH0180	*Dilophotes*	AT	AT	AT	-	Sumatra Utara, Brastagi, Gn. Sibayak
UPOLTH0183	*Dilophotes*	AT	AT	-	AT	Sumatra, Utara, Brastagi, Gn. Sibayak
UPOLTH0127	*Dilophotes*	AU	AU	AU	AU	Philippines, Mindanao, Bagongsilang
UPOLTH0128	*Dilophotes*	AU	AU	AU	AU	Philippines, Mindanao, Bagongsilang
UPOLTH0052	*Dilophotes*	AV	AV	AV	-	Java, Barat, Puncak Pass, Cipanas
UPOLTH0053	*Dilophotes*	AV	AV	AV	-	Java, Barat, Puncak Pass, Cipanas
UPOLTH0055	*Dilophotes*	AW	AWa	AW	-	Sumatra, Jambi, Kersik Tua

UPOLTH0056	*Dilophotes*	AW	AWb	AW	-	Sumatra, Jambi, Kersik Tua
UPOLTH0057	*Dilophotes*	AW	AWb	AW	-	Sumatra, Jambi, Gn. Kerinci
UPOLTH0076	*Dilophotes*	AW	AWb	AW	AC	Sumatra, Jambi, Gn. Kerinci
UPOLTH0126	*Dilophotes*	AU	AU	AU	-	Philippines, Mindanao, Bagongsilang
UPOLTH0125	*Dilophotes*	AX	AX	AX	AX	Philippines, Mindanao, Bagongsilang
UPOLTH0124	*Dilophotes*	AY	AY	AY	AY	Philippines, Mindanao, Bagongsilang
UPOLTH0051	*Dilophotes*	AZ	AZ	AZ	-	Sumatra, Jambi, Kersik Tua, Gn. Tujuh
UPOLTH0035	*Dilophotes*	BA	BA	BA	-	Borneo, Sabah, Gn. Emas
UPOLA00001	*Dilophotes*	BB	-	BBa	BBa	Sumatra, Utara, Brastagi, Gn. Sibayak
UPOLTH0155	*Dilophotes*	BB	BBb	BBb	-	Malay Peninsula, Johor, Kota Tinggi
UPOLTH0157	*Dilophotes*	BB	BBb	BBb	-	Malay Peninsula, Johor, Kota Tinggi
UPOLTH0159	*Dilophotes*	BB	BBb	BBb	BBb	Malay Peninsula, Pahang, Tanah Rata

Supplementary Table S4. Primers used for PCR amplifications and sequencing.

Fragment	Code	-mer	Sequence (5’ >> 3’)
16S rDNA	16a	20	CGCCTGTTTAACAAAAACAT
	16b	22	CCGGTCTGAACTCAGATCATGT
	ND1A	27	GGTCCCTTACGAATTTGAATATATCCT
cox1 mtDNA	JerM	23	CAACAYYTATTTTGRTTYTTTGG
	Pat	25	TCCATTGCACTAATCTGCCATATTA
	Marilyn	21	TCATAAGTTCAGTATCATTG
	Marcy	27	TARTTCRTATGWTCAATAYCAYTGRTG
nad5 mtDNA	OF1	29	CCTACTCCTGTTTCTGCTTTAGTTCATTC
	R6	29	GAAACGAAAAATCGTATTTAATTTCGACT

Supplementary Table S5. The length of DNA fragments, the numbers of informative characters in datasets, and partitions.

Datasets/Partitions	All data	*coi*+tRNA+coii**	*rrnl+tRNA+nad1**	*nad5*+tRNAs*
# of specimens	225	194	194	129
BlastAlign				
# of characters	3318	1103	912	1303
parsimony inf.	1791	615	355	821
Mafft				
# of characters	3170	1102	811	1257
parsimony inf.	1824	601	359	864

* the protein coding were additionally partitioned by codon position.

Supplementary Table S6. The node probability values for the reconstruction of the evolution of the monomorphic and dimorphic aposematic patterns. Tree nodes are defined in the Figure S6.

Tree node	Monomorphic	Dimorphic
23	0.9836	0.0164
34	0.8933	0.1067
35	0.0643	0.9357
48	0.9876	0.0124
49	0.8590	0.1410
50	0.8560	0.1440
51	0.8502	0.1498
52	0.8527	0.1473
53	0.0708	0.9292

Supplementary Table S7. The node probability values for the reconstruction of uniform and bicolored elytra. Tree nodes are defined in the Figure S6.

Tree node	Elytra:	
	Uniformly coloured	Bright & black coloured
5	0.8105	0.1895
7	0.9867	0.0133
8	0.978	0.0220
21	0.9761	0.0239
23	0.0637	0.9363
24	0.9849	0.0151
25	0.9879	0.0121
26	0.9870	0.0130
27	0.9677	0.0323
28	0.2157	0.7843
29	0.2217	0.7783
31	0.9867	0.0133
32	0.9673	0.0327
37	0.0127	0.9873
55	0.0210	0.9790
58	0.0253	0.9747

Supplementary Table S8. The node probability values for the reconstruction of individual aposematic patterns. Tree nodes are defined in the Figure S6.

Tree node	black	red	red & black	striped	yell.&black	yellow	sld1	sld2	yellow humeri
1	0.8684	0.0388	0.0049	0.0013	0.0028	0.0819	0.0008	0.0005	0.0005
2	0.9853	0.0024	0.0028	0.0004	0.0015	0.0072	0.0001	0.0003	0
3	0.8683	0.0383	0.0044	0.0011	0.0025	0.0841	0.0007	0.0004	0.0003
4	0.8684	0.0307	0.0051	0.0023	0.0027	0.0888	0.0012	0.0005	0.0004
5	0.8258	0.0101	0.0167	0.1131	0.0069	0.0191	0.0039	0.0024	0.0021
6	0.8244	0.0271	0.0059	0.0037	0.004	0.13	0.002	0.0013	0.0016
9	0.8422	0.0545	0.0047	0.0008	0.0031	0.0935	0.0007	0.0004	0.0003
10	0.7438	0.1163	0.0071	0.0004	0.0041	0.1268	0.0005	0.0005	0.0005
11	0.5275	0.0475	0.006	0.0005	0.0029	0.4127	0.0008	0.0005	0.0015
12	0.5206	0.0295	0.0076	0.0015	0.0049	0.4318	0.0012	0.0008	0.0021
13	0.0285	0.0045	0.0017	0.009	0.0009	0.9607	0.0005	0.0007	0.0015
14	0.2325	0.7055	0.0225	0.0004	0.0071	0.0291	0.0019	0.0007	0.0004
15	0.1353	0.8151	0.0183	0.0005	0.0056	0.0213	0.0027	0.0007	0.0005
16	0.1442	0.7888	0.0383	0.0001	0.0175	0.0087	0.0023	0.0001	0
17	0.0912	0.8424	0.0192	0.0003	0.0101	0.0347	0.0013	0.0003	0.0005
18	0.0037	0.0115	0.0015	0.0001	0.0003	0.9812	0.0003	0.0005	0.0009
19	0.0011	0.9969	0.0017	0 0	0.0001	0.0001	0	0	0
23	0.0041	0.3474	0.3899	0.0029	0.0067	0.0005	0.2462	0.0007	0.0015
24	0.1496	0.764	0.0548	0.0003	0.0239	0.0035	0.004	0	0
25	0.1541	0.7646	0.0491	0.0001	0.0256	0.002	0.0045	0	0
26	0.6674	0.2351	0.0444	0.0001	0.0576	0.002	0.0039	0.0003	0.0001
27	0.7084	0.1582	0.0449	0.0007	0.0996	0.0037	0.004	0.0004	0.0004
28	0.1312	0.0015	0.004	0.0001	0.8626	0.0001	0.0004	0.0001	0
29	0.1356	0.0008	0.0033	0.0001	0.8594	0.0001	0.0004	0.0003	0
30	0.0748	0.0014	0.0043	0.0004	0.844	0.0003	0.0007	0	0.0741
31	0.0813	0.8452	0.0396	0.0003	0.024	0.0013	0.0081	0	0.0001
32	0.0703	0.8442	0.0391	0.0004	0.0261	0.0015	0.0181	0.0001	0.0003
34	0.0193	0.1473	0.0525	0.0035	0.1657	0.0025	0.6062	0.0011	0.0019
35	0.0011	0.0095	0.0065	0.0012	0.0128	0.0005	0.9683	0	0.0001
37	0.0496	0.0432	0.8004	0.0031	0.0759	0.0011	0.0264	0.0003	0.0001

38	0.0424	0.0131	0.8611	0.0031	0.0527	0.0005	0.0268	0.0003	0.0001
35	0.0144	0.0057	0.8576	0.0885	0.0179	0.0007	0.0144	0.0004	0.0004
43	0.0413	0.0056	0.8764	0.0011	0.0468	0	0.0283	0.0004	0.0001
44	0.034	0.0016	0.9164	0.0004	0.0388	0	0.0087	0.0001	0
45	0.0521	0.0005	0.8824	0	0.0623	0	0.0025	0.0001	0
46	0.0439	0.0004	0.8859	0	0.0673	0	0.0024	0.0001	0
49	0.0483	0.0033	0.8016	0.0011	0.0484	0.0004	0.0948	0.002	0.0001
50	0.0207	0.0019	0.7328	0.0011	0.0459	0.0001	0.1925	0.0049	0.0001
51	0.0083	0.0013	0.6994	0.0007	0.0443	0	0.2452	0.0008	0.0001
52	0.008	0.0024	0.5211	0.0013	0.1352	0.0009	0.3297	0.0007	0.0007
53	0.0067	0.0007	0.7008	0.0007	0.0369	0.0001	0.2528	0.0005	0.0007
54	0.0007	0.0008	0.0705	0.0007	0.0051	0	0.9219	0.0001	0.0003
56	0.0008	0.0148	0.9825	0.0001	0.0008	0	0.0009	0	0
59	0.0195	0.0003	0.9801	0.0001	0	0	0	0	0

Designation of colour patterns as in Figure 1:
black – the uniformly black upper side of the body, i.e., the pronotum and elytra
red – the uniformly dark red upper side of the body
yellow – the uniformly yellow upper side of the body
striped – the black pronotum, striped humeri and the black apical part of the elytra
yellow humeri – the dark brown upper side of the body with light brown humeri
yellow & black – the black or yellow pronotum, yellow humeri to basal 2/3 of the elytra and the black posterior part of elytra
red & black – the black pronotum, orange to red humeri and the black posterior part of the elytra
sld1 – the male yellow and black coloured, female black with the red humeri or the basal part of elytra (as the type red/black above)
sld2 – the male uniformly black, female black with the light brown basal part of the elytral costae (Fig. S1A–N)

Supplementary Table S9. The node probability values for the reconstruction of the ancestral areas.

Tree node	Java	Philippines	Japan	Laos, Thail.*	Sumatra	Malaya	China**	India	Borneo
1	0.0005	0.0005	0.0005	0.8003	0.0804	0.0991	0.0139	0.0003	0.0045
2	0.0031	0.0012	0.0031	0.759	0.0256	0.0207	0.1793	0.0035	0.0047
3	0.0003	0.0003	0.0003	0.8032	0.0811	0.0999	0.0115	0	0.0036
4	0.0012	0.0015	0.0005	0.7355	0.1036	0.1389	0.1389	0.0003	0.0068
5	0.0008	0.0003	0.0007	0.0109	0.0156	0.9685	0.0009	0.0003	0.002
6	0.0028	0.0033	0.0007	0.7076	0.1361	0.127	0.0124	0.0009	0.0091
7	0.0007	0.0007	0	0.0109	0.9903	0.0096	0.0003	0.0001	0.0025
8	0.0003	0.0007	0	0.0077	0.9963	0.0007	0.0001	0.0001	0.0017
9	0.0001	0.0003	0.0001	0.812	0.0765	0.0972	0.0099	0.0001	0.0037
10	0	0	0	0.8294	0.0737	0.0844	0.0088	0.0001	0.0036
11	0.0005	0.0003	0.0003	0.8227	0.0779	0.0851	0.0088	0.0001	0.0044
12	0.0016	0.0019	0.0012	0.7756	0.0684	0.131	0.012	0.0017	0.0065
14	0.0005	0	0	0.8454	0.074	0.0663	0.01	0.0001	0.0037
15	0.0009	0.0003	0.0003	0.8848	0.0507	0.048	0.01	0.0007	0.0044
16	0.0005	0.0007	0.0005	0.4883	0.3276	0.0915	0.0677	0.0019	0.0213
17	0.0005	0.0009	0.0048	0.5861	0.2153	0.048	0.1149	0.0061	0.0233
18	0.002	0.0031	0.0036	0.751	0.0649	0.0261	0.0472	0.0893	0.0128
19	0.0007	0.0013	0.0139	0.5117	0.1906	0.0347	0.2156	0.0017	0.0299
20	0.0004	0.0007	0.0075	0.5081	0.1898	0.0311	0.2305	0.0015	0.0305
21	0.0001	0.0001	0.0013	0.0912	0.0301	0.0048	0.8664	0.0005	0.0053
22	0.0004	0.0011	0.0041	0.5337	0.214	0.0313	0.1717	0.0013	0.0424
24	0.0004	0.0007	0.0003	0.2632	0.5289	0.1181	0.0584	0.0011	0.0291
25	0.0005	0.0012	0.0001	0.2334	0.5555	0.0988	0.0703	0.0007	0.0395
26	0.0009	0.0157	0.0009	0.137	0.5523	0.214	0.0427	0.0007	0.0357
27	0.0009	0.0076	0.0004	0.0672	0.5559	0.3118	0.0205	0.0003	0.0353
28	0.0001	0.0005	0.0003	0.0049	0.6724	0.2694	0.0008	0	0.0515
29	0.0003	0.0005	0.0001	0.0025	0.5251	0.3625	0.0001	0.0003	0.1085
30	0.0001	0	0	0	0.9903	0.0075	0	0	0.0021

31	0.0009	0.0009	0.0004	0.2338	0.4921	0.0578	0.1389	0.0007	0.0744
32	0.0012	0.0016	0.0007	0.2377	0.4733	0.0243	0.1414	0.0007	0.1192
33	0.0011	0.0011	0.0015	0.5421	0.0674	0	0.3734	0.0011	0.0125
34	0.0007	0.0007	0	0.0152	0.1868	0	0.0063	0.0003	0.7902
35	0.0012	0.0007	0	0.0096	0.1901	0.0003	0.0035	0	0.7946
37	0.002	0.0001	0	0.0052	0.8491	0.1373	0.0013	0	0.0049
38	0.0021	0.0003	0	0.0016	0.8492	0.1421	0.0004	0	0.0043
39	0.0867	0.0015	0.0007	0.0028	0.3186	0.5838	0.0011	0.0001	0.0048
40	0.0007	0	0	0	0.0101	0.9888	0	0.0001	0.0003
42	0	0	0	0	0.0065	0.9935	0	0	0
43	0.0017	0.0001	0.0001	0.0004	0.8662	0.1274	0.0001	0	0.004
44	0.0183	0.0009	0.0001	0.0013	0.8322	0.133	0.0003	0.0005	0.0133
45	0.0012	0	0.0001	0.0001	0.8062	0.152	0	0	0.0405
46	0.0008	0	0.0001	0	0.8259	0.1199	0	0	0.0535
47	0.0001	0.0001	0.0001	0	0.9988	0.0009	0	0	0.0003
48	0.0001	0.0001	0.0001	0	0.0116	0.0013	0	0	0.9871
49	0.0004	0.0003	0.0001	0.0001	0.88	0.1152	0	0.0001	0.0037
50	0.0001	0.0004	0.0001	0	0.9375	0.0572	0	0	0.0049
51	0.0001	0.0005	0.0001	0.0001	0.9715	0.0264	0	0	0.0015
52	0.0001	0.0115	0.0001	0.0004	0.9633	0.0221	0.0004	0.0004	0.0021
53	0.0001	0.0011	0.0001	0.0001	0.9745	0.0233	0	0	0.0008
54	0.0103	0.0007	0.0001	0.0001	0.9637	0.0224	0.0001	0.0001	0.0025
55	0.0001	0.0077	0	0.0001	0.9699	0.0203	0	0	0.0019
56	0	0.9867	0.0001	0	0.0128	0.0003	0	0	0.0001
57	0	0.0007	0	0	0.9779	0.0193	0	0	0.0021
58	0.0001	0.0003	0	0.0001	0.9727	0.018	0	0	0.0088
59	0	0	0	0.0001	0.9383	0.0612	0	0	0.0004
60	0.0009	0.0017	0.0015	0.133	0.5401	0.0371	0.0443	0.0012	0.2402

* China including Taiwan and Hainan
** Indo-Burma: Laos, Thailand, Cambodia, Vietnam, Burma.

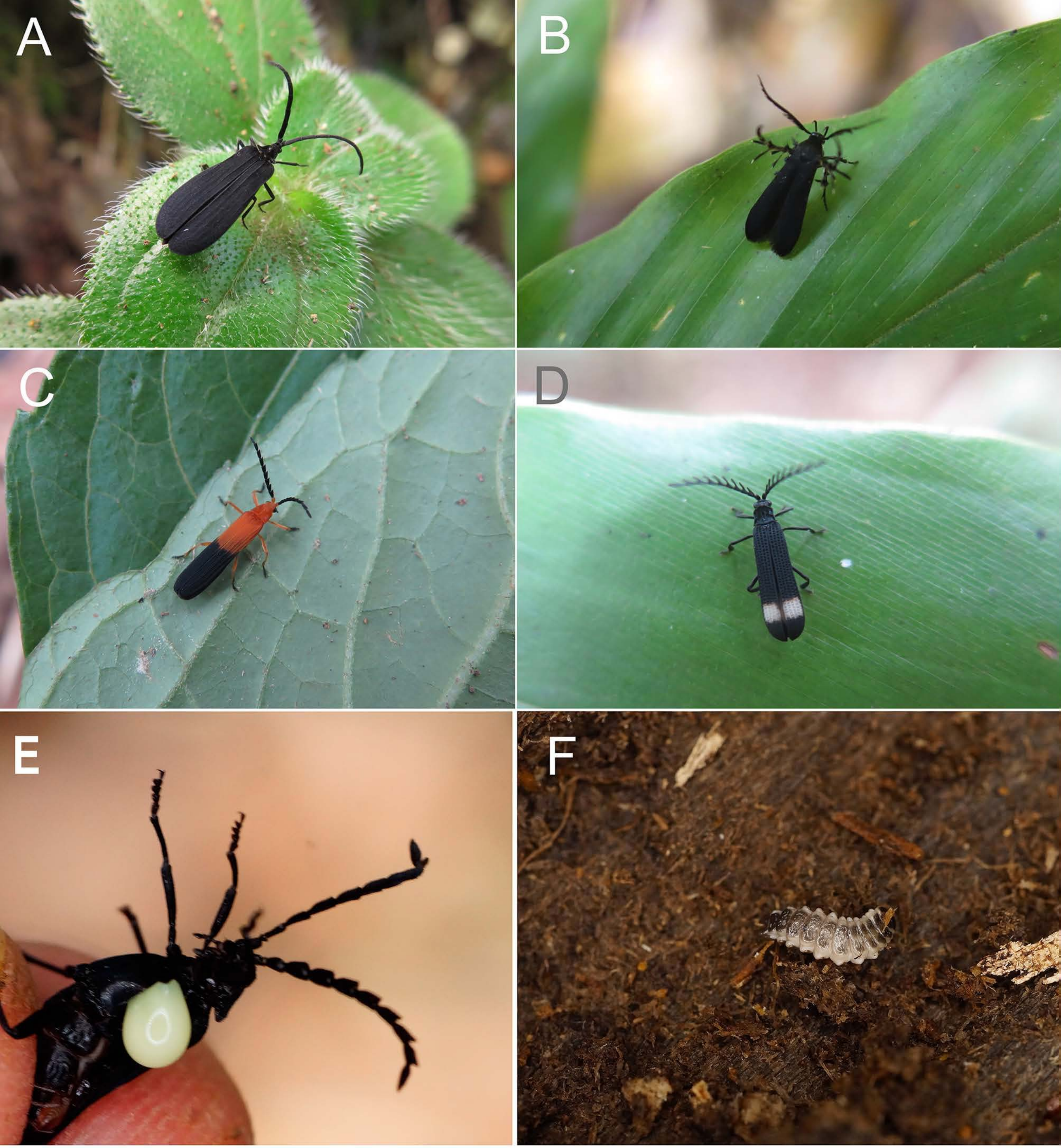

Figure S1. (A) *Lyropaeus* sp.; (B) Syntopically occurring moth; (C–D) Syntopically occurring mimetics from New Guinea as an example of multiple patterns pruduced by net-winged beetles in a single community; (E) Haemolymph produced by a disturbed net-winged beetle; (F) larva of a net-winged beetle. (c) Authors.

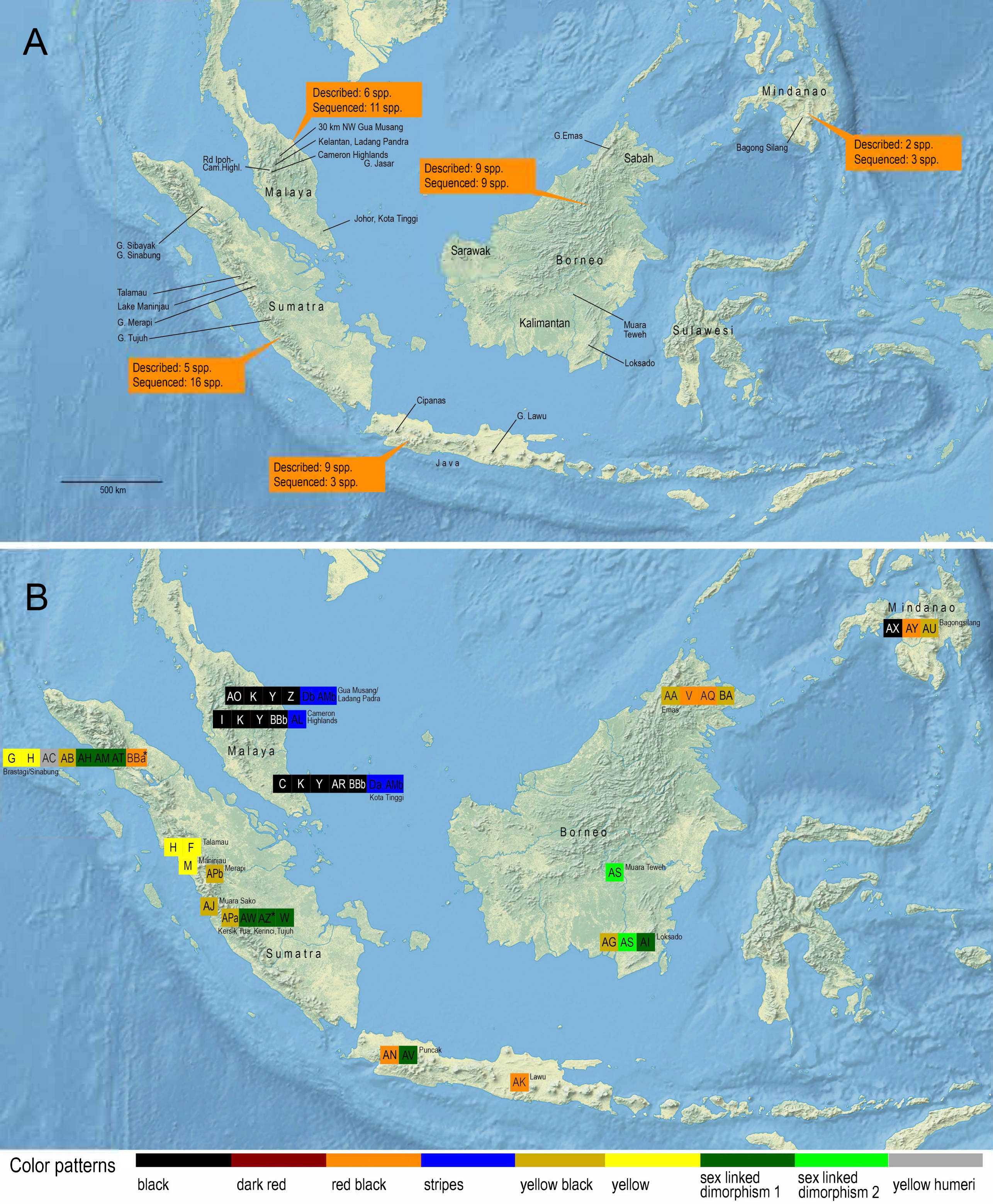

Supplementary Figure S2. (A) Sampled localities in Malaya, the Sundaland, and Philippines with the numbers of formally described and recorded species. (B) Distribution of color patterns and species in Malaya, the Sundaland, and Philippines.

Map was downloaded from Natural Earth server (http://www.naturalearthdata.com) and edited using Adobe Photoshop CS6 (http://www.adobe.com/products/photoshop.html).

*If only one sex is available and individual(s) resemble(s) monomoprhic and dimorphic pattern, then a monomorphic patters is assigned.

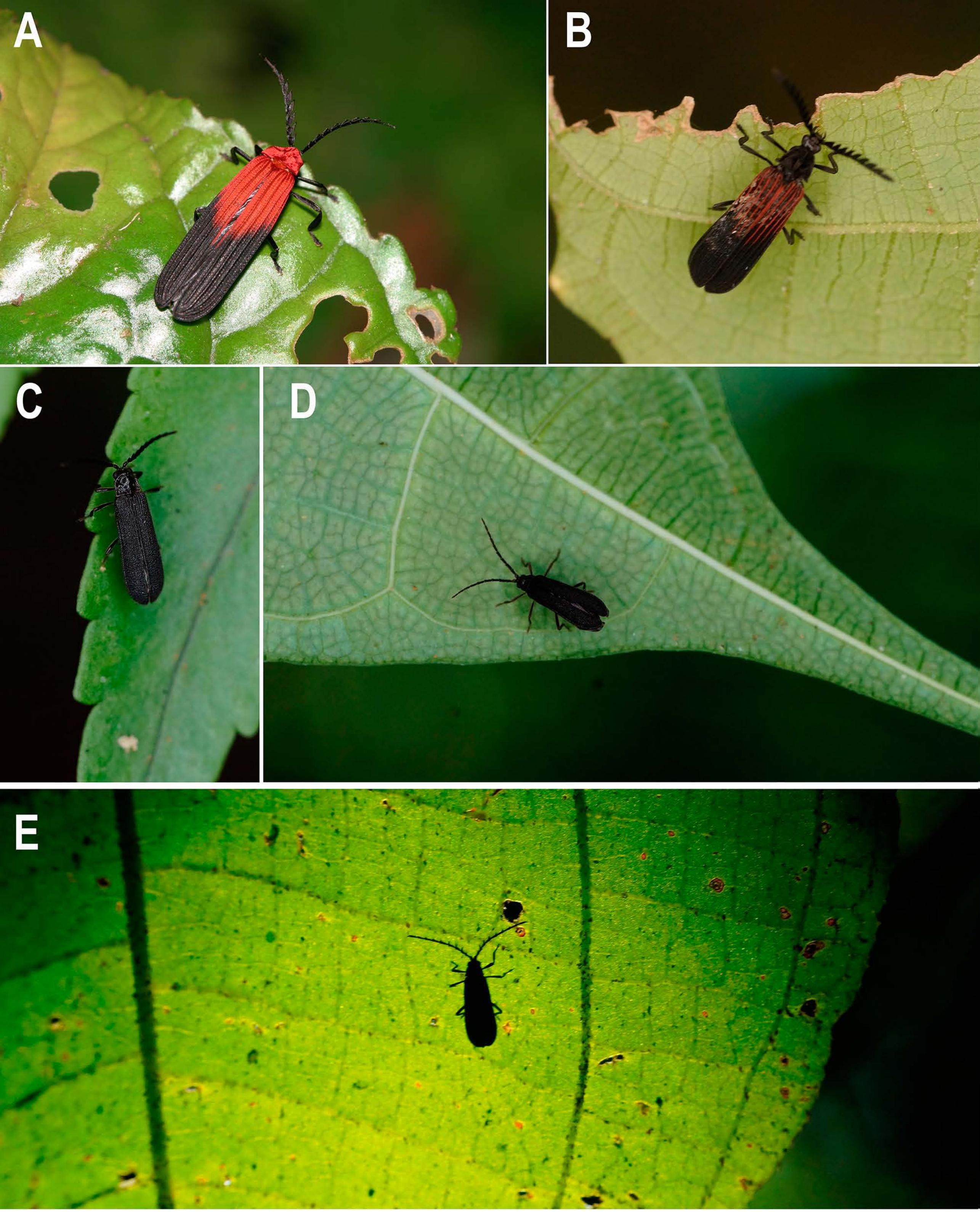

Supplementary Figure S3. Colour patterns of net-winged beetles in the Sundaland. (A) *Cautires* sp., Sumatra; (B) *Plateros* sp., Malay Peninsula; (C) *Plateros* sp., Malay Peninsula; (D) *Libnetis* sp., Malay Peninsula; (D) *Dihammatus* sp., Malay Peninsula.
The figure 2D shows the individual sitting on the bottom side of the leave when light comes from above and the size and shape are principal traits observed by potential predators. (c) Authors.

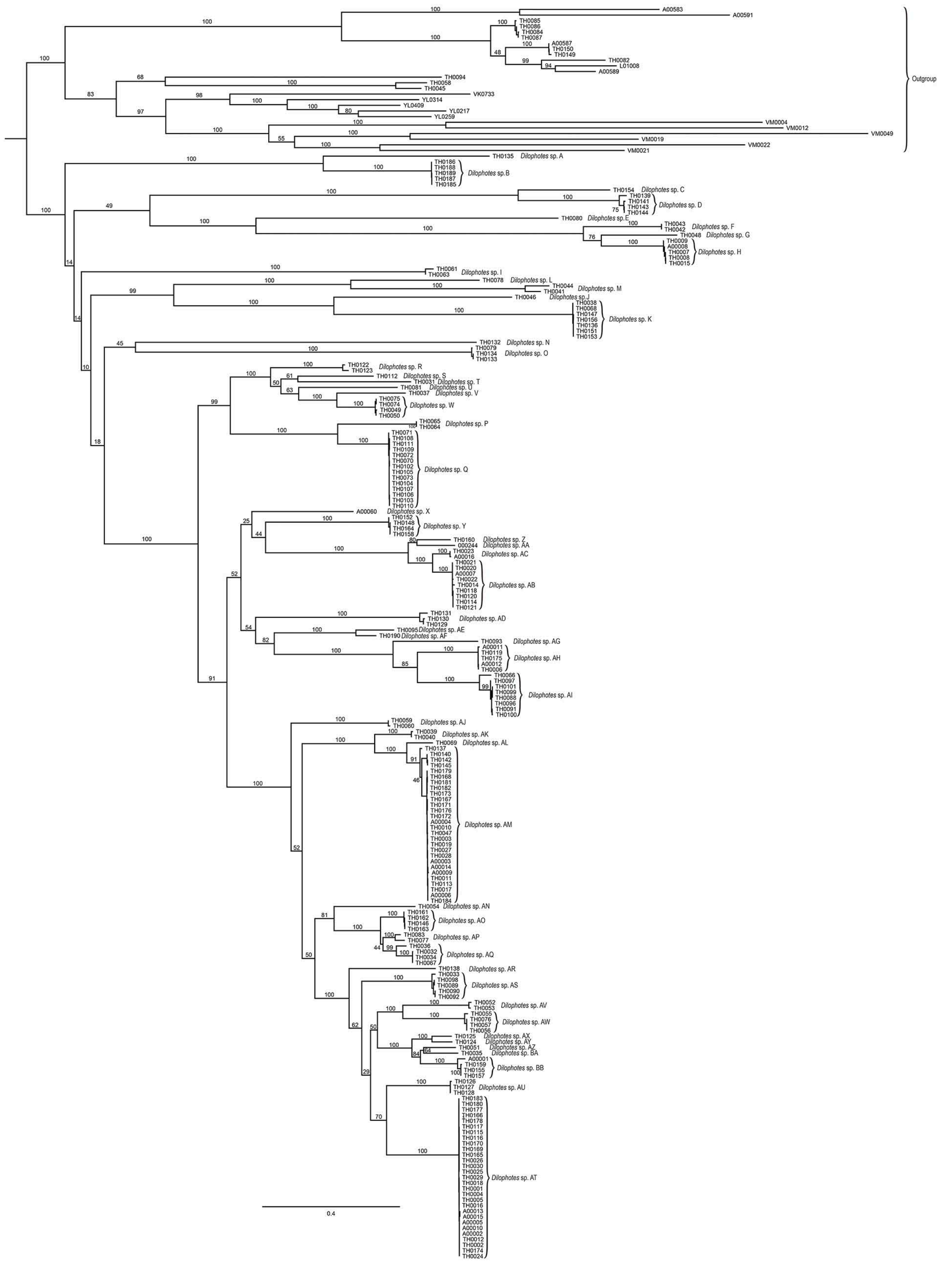

Supplementary Figure S4. The maximum likelihood phylogenetic hypothesis of *Dilophotes* inferred from the MAFFT aligned dataset. The numbers above branches show bootstrap supports.

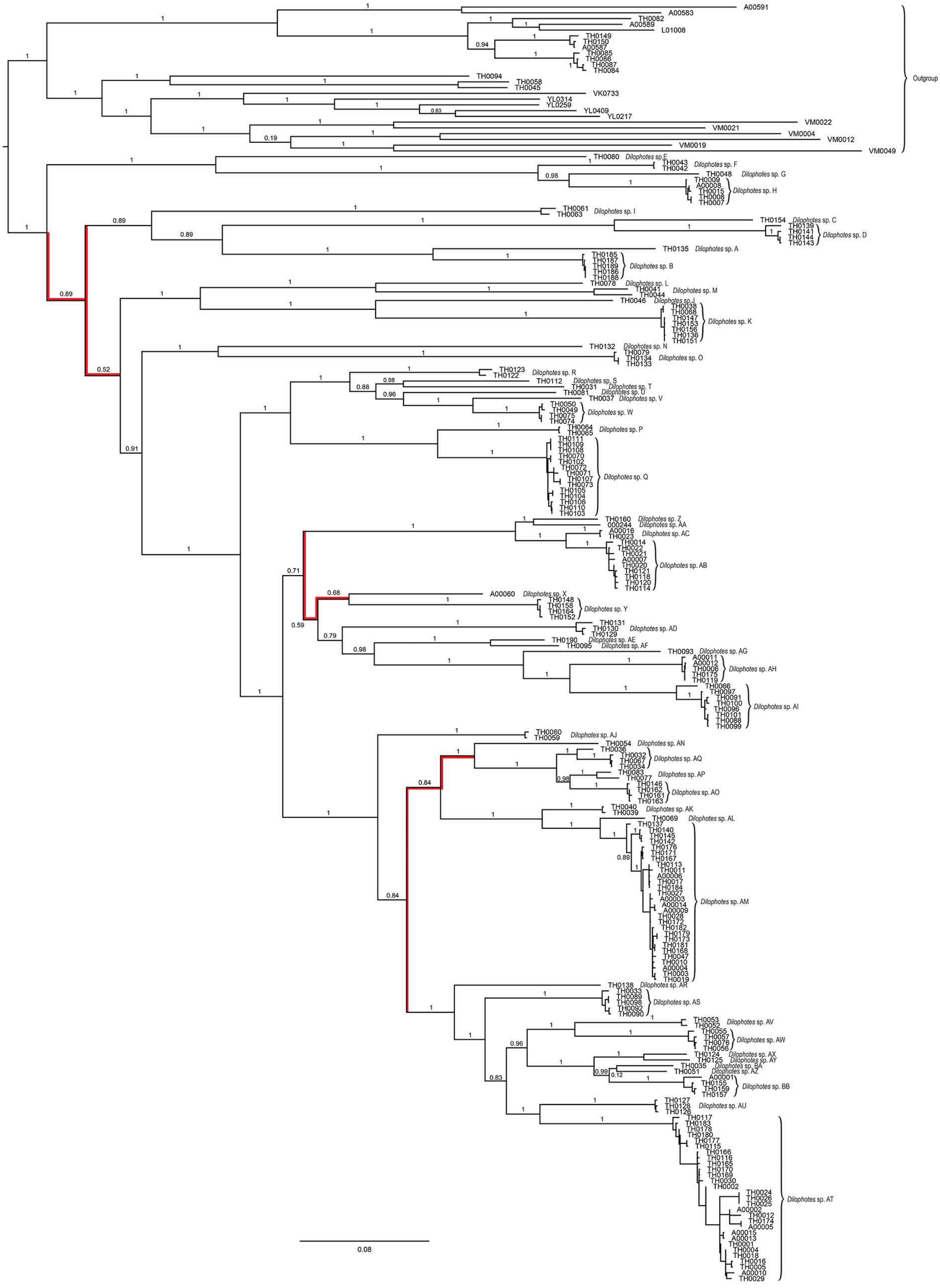

Supplementary Figure S5. The Bayesian phylogenetic hypothesis of *Dilophotes* inferred from the MAFFT aligned dataset. The numbers above branches show posterior probabilities. The branches absent in the maximum likelihood topology are marked in red.

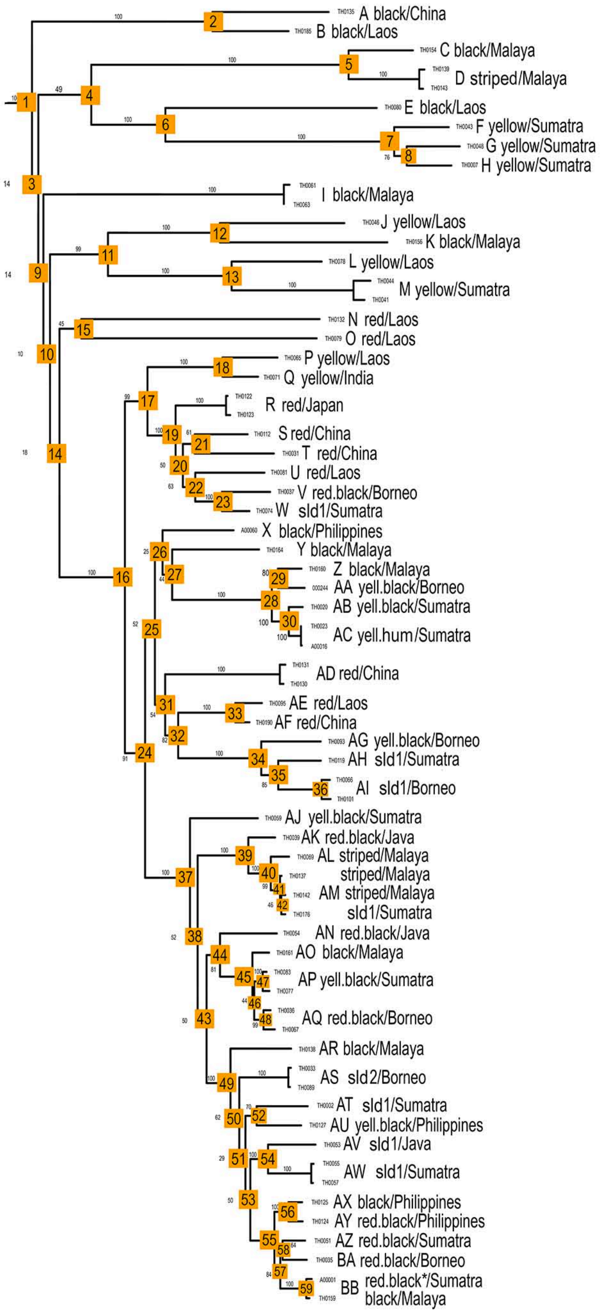

Supplementary Figure S6. The numbers designating splits in the pruned tree. The outgroups and multiple specimens of a species are pruned out. The tree was produced by maximum likelihood analysis of the MAFFT alignment of 196 taxa and 29 outgroups.

*If only one sex is available and individual(s) resemble(s) monomorphic and dimorphic pattern, then a monomorphic patters is assigned

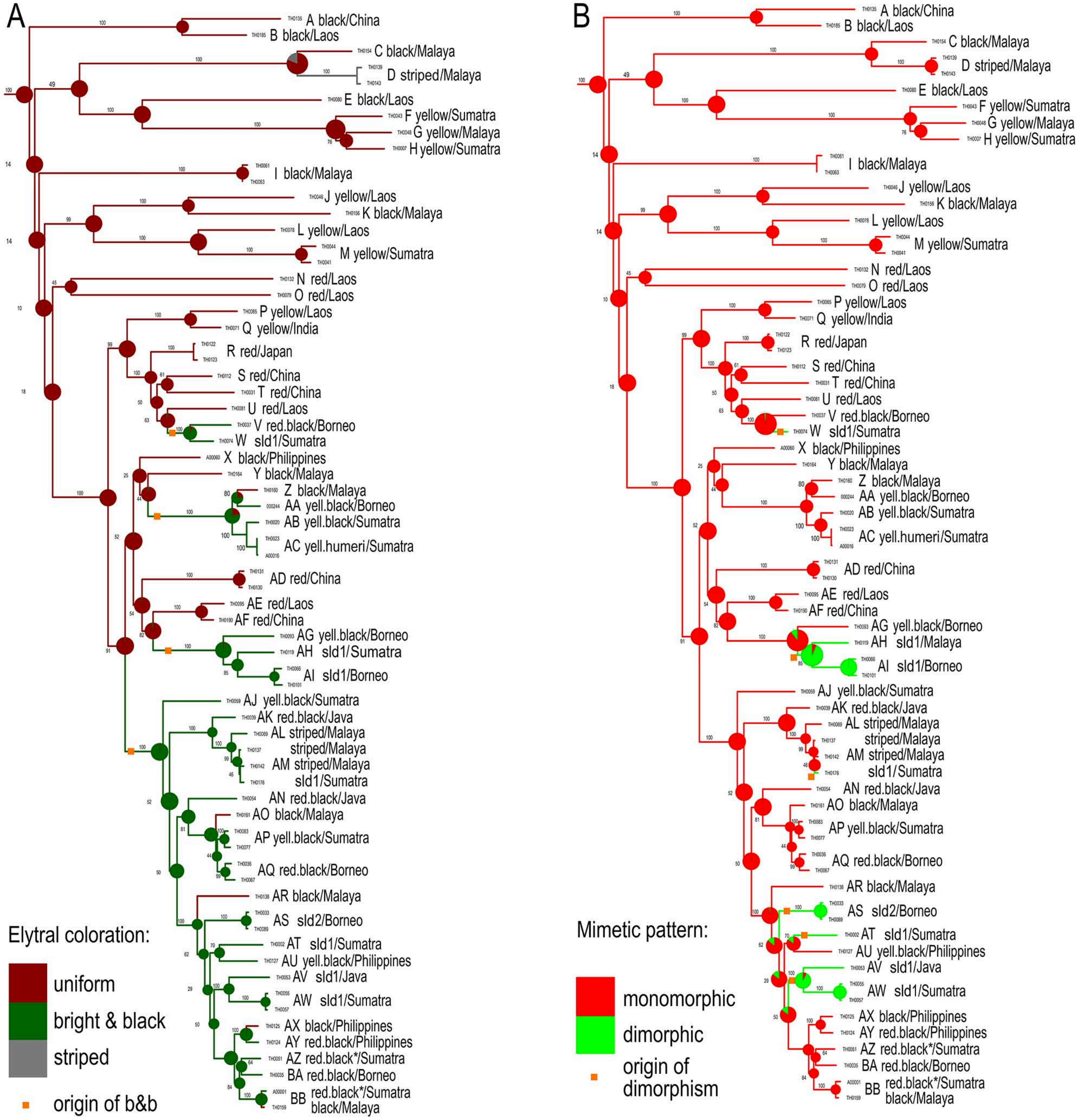

Supplementary Figure S7. The Bayesian phylogenetic reconstruction of ancestral states.
(A) uniform, striped, and bright & black elytral patterns;
(B) sexually monomorphic and dimorphic aposematic patterns, i.e. both sexes similarly coloured or males yellow/black and females red/black and females striped and males black, respectively.

*If only one sex is available and individual(s) resemble(s) monomorphic and dimorphic pattern, then a monomorphic pattern is assigned
